# SUMO E3 ligase SIZ1 promotes nuclear condensate-mediated immune activation in Arabidopsis

**DOI:** 10.1038/s41467-026-72063-x

**Published:** 2026-04-15

**Authors:** Min Jia, Enrico Calvanese, Rachel Bai, Yiling Fang, Yangnan Gu

**Affiliations:** Department of Plant and Microbial Biology, University of California, Berkeley, CA USA

**Keywords:** Plant immunity, Plant signalling, Ligases

## Abstract

SIZ1, a major plant SUMO E3 ligase, has diverse roles in development and immunity. Although loss-of-function phenotypes have categorized SIZ1 as a negative immune regulator, its broad substrate range complicates interpretation of its core function. Here we show that SIZ1 overaccumulation unexpectedly activates robust immune responses and cell death, dependent on its E3 ligase activity. Proximity-labeling proteomics revealed that SIZ1 function converges on the MOS4-Associated Complex (MAC), a critical immune signaling module that forms pro-immune nuclear condensates during pathogen attack. Both SIZ1 and the immune receptor SNC1 are recruited to MAC-dependent nuclear condensates (MDNCs) upon pathogen challenge, where they synergistically potentiate immune responses and cell death. SIZ1 SUMOylates and stabilizes MAC components, reinforcing MDNC formation and sustaining immune signaling. This mechanism counteracts the previously identified MDNC-inhibitory mechanism mediated by karyopherin KA120, establishing a regulatory system that balances immune-promoting condensate assembly with disassembly to prevent autoimmunity while maintaining rapid defense capacity.

## Abstract (web summary)

SIZ1 promotes plant immunity by SUMOylating MOS4-Associated Complex components, reinforcing pro-immune nuclear condensates during pathogen attack and balancing robust defence with autoimmunity prevention.

## Introduction

Plants have evolved sophisticated immune systems that maintain a delicate balance between robust pathogen defense and optimal physiological fitness when thriving in pathogen-rich environments. The plant immune system functions through two interconnected tiers: pattern-triggered immunity (PTI), which is mediated by cell-surface receptors recognizing conserved microbial signatures, and effector-triggered immunity (ETI), which is activated when intracellular NLR (Nucleotide-binding Leucine-rich Repeat) receptors detect pathogen effector proteins^1–3^. PTI establishes basal resistance through processes including reactive oxygen species production and callose deposition, whereas ETI triggers more robust defense outputs, including hypersensitive cell death and systemic acquired resistance. These systems demonstrate synergistic interactions, with PTI potentiating ETI responses and ETI providing feedback enhancement to PTI through substantial transcriptional reprogramming^4,5^.

The emerging concept of biomolecular condensates has introduced a novel dimension to our understanding of immune signaling regulation through spatial compartmentalization. Accumulating evidence demonstrates that many plant immune proteins undergo liquid-liquid phase separation to form dynamic, membrane-less condensates that spatially organize and activate signaling modules^6–13^. For instance, Toll/Interleukin-1Receptor (TIR) domain-containing NLR (TNL) proteins assemble into substrate-induced condensates both in vitro and in planta, with disruption of these condensates significantly impairing TNL cell death activity^7^. Similarly, MAC3, a core subunit of the conserved spliceosome-associated MAC complex, forms MAC3-dependent nuclear condensates (MDNCs) following pathogen perception. These MDNCs sequester negative immune regulators to promote immune activation, functioning as essential components downstream of TNL-mediated ETI^14,15^. However, whether TNL condensates and MDNCs represent identical or distinct condensate populations, and their functional relationships, remain to be elucidated.

The mechanisms conferring pathogen resistance can lead to autoimmunity when inadequately regulated, resulting in growth defects and spontaneous cell death in pathogen-free conditions^16^. Accumulating evidence underscores the critical role of nuclear transport machinery in maintaining immune homeostasis. Specifically, karyopherin family members—nuclear transport receptors responsible for translocating macromolecules across the nuclear envelope—have proven essential for preventing autoimmune activation^14,17–22^. Among these, KA120 constrains the nuclear activity of the TNL protein SNC1^19^ and directly interacts with MAC3 to inhibit MDNC condensate formation and MDNC-dependent immune activation^14^. This latter function exemplifies a noncanonical chaperoning activity of karyopherins independent of their nucleocytoplasmic transport function, which appears evolutionarily conserved across eukaryotes. In humans, karyopherin importin-β2 inhibits pathological condensate formation of the mRNA-binding protein FUS, thereby preventing cytoplasmic granule accumulation associated with neurodegenerative diseases^23–26^.

Plant immune activation also undergoes fine-tuning through diverse post-translational modifications (PTMs), with SUMOylation, the covalent attachment of Small Ubiquitin-like Modifier (SUMO) proteins, emerging as a critical regulatory mechanism^27^. In plant cells, SUMOylation is catalyzed by a conserved enzymatic cascade involving an E1 activating enzyme (SAE1–SAE2), a single E2 conjugating enzyme (SCE1), and a small set of E3 ligases (SIZ1, MMS21, PIAL1, and PIAL2), which determine substrate specificity^28–30^. In *Arabidopsis*, SIZ1 is the predominant SUMO E3 ligase responsible for conjugating SUMO proteins to hundreds of substrates^31,32^. Loss-of-function mutations in *SIZ1* result in pleiotropic phenotypes, including autoimmune induction and increased sensitivity to various abiotic stresses such as cold, drought, and nutrient deficiency^29,31,33–36^. Moreover, SIZ1 governs the trade-off between immunity and growth, particularly through modulation of SNC1 and transcriptional co-repressor TPR1 activity^37–40^. Two aphid and oomycete effectors were reported to target SIZ1, but paradoxically increasing SIZ1 protein levels and E3 ligase activity, respectively^41^. Despite these advances, the intersection of SUMOylation with karyopherin proteins and nuclear condensate dynamics in orchestrating immune responses remains unexplored.

In this study, we demonstrate that the SUMO E3 ligase SIZ1 regulates immune activation in *Arabidopsis* through its involvement in KA120-chaperoned MAC nuclear condensates. We showed that ectopic expression of SIZ1 unexpectedly triggers robust immune responses and cell death in a manner dependent on its E3 ligase activity. Through proximity labeling proteomics, we identified the MAC complex as a shared substrate hub of both SIZ1 and karyopherin KA120, suggesting their functional convergence on MAC-mediated immune signaling. We further reveal that both SIZ1 and the TNL immune receptor SNC1 are recruited into pathogen-induced MAC condensates, where they synergistically potentiate immune responses and cell death. SIZ1 SUMOylates and stabilizes MAC components, thereby reinforcing MDNC formation and sustaining immune signaling. In contrast, KA120 overexpression disrupts MAC condensate formation and suppresses SIZ1-dependent immune induction, underscoring antagonistic regulatory mechanisms that coordinate MDNC dynamics. Collectively, our findings reveal a previously uncharacterized positive immune regulatory role of SIZ1 and provide a mechanistic framework linking SUMO modification, MAC stability, and nuclear condensates in plant immune regulation.

## Results

### *SIZ1* and *KA120* display genetic interactions

The *siz1-2* loss-of-function mutant displays a phenotype remarkably similar to the *ka120* mutant, not only in terms of autoimmune phenotypes (Supplementary Fig. 1a), but also in enhanced sensitivity to multiple abiotic stresses. Both mutants exhibit hypersensitivity to abscisic acid (ABA) and the DNA damage (Supplementary Fig. 1b, c), traits uncommonly observed in other autoimmune mutants. Moreover, loss of *SNC1*, a TNL gene, partially suppresses the autoimmune phenotype in both *siz1* and *ka120* mutants^19,37^. These phenotypic similarities suggest functional connections between SIZ1 and KA120.

**Fig. 1:**
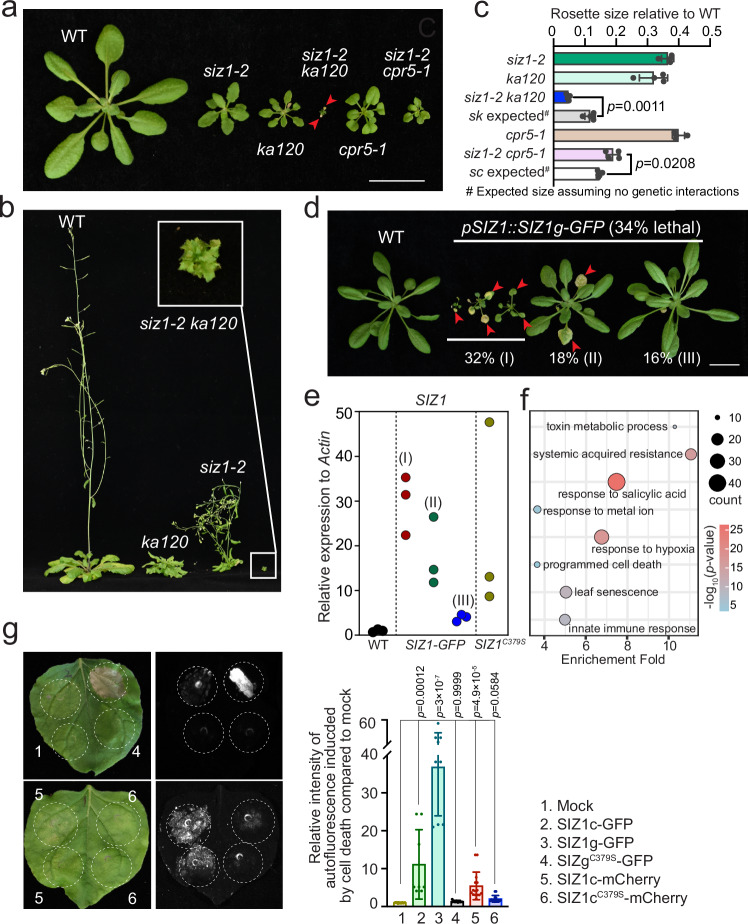
*SIZ1* genetically interacts with *KA120* and plays a dual role in immune and cell death regulation. **a**, **b** Four-week-old (**a**) and eight-week-old (**b**) soil-grown plants of indicated genetic backgrounds. Scale bar, 2 cm. Similar results were obtained in three independent experiments. **c** Quantification of rosette size in indicated mutants relative to WT plants. Data are presented as mean ± SD (*n* = 4 for each genetic background, two-tailed *t*-test, *p* < 0.01). Similar results were obtained in three independent experiments. The expected rosette size for the *siz1-2 ka120* (*sk*) and *siz1-2 cpr5-1* (*sc*) double mutants under the null hypothesis (no genetic interaction) was calculated by multiplying the size effects of the corresponding single mutants. **d** Five-week-old soil-grown plants of WT and independent *pSIZ1::SIZ1g-GFP* of T1 lines, grouped according to phenotype severity. Percentages indicate the proportion of each category among the total recovered transgenic lines. Scale bar, 2 cm. **e** RT-qPCR analysis of *SIZ1* expression in 3-week-old *pSIZ1::SIZ1g-GFP* transgenic lines, normalized to WT. Data points represent three independent transgenic lines, colored according to their phenotype category. **f** Gene ontology (GO) analysis of up-regulated differentially expressed genes (DEGs) in *pSIZ1::SIZ1g-GFP* transgenics with autoimmune phenotypes. GO enrichment analysis was performed using the enrichGO function from the clusterProfiler R package. The statistical significance of enrichment for each GO term was assessed using a hypergeometric test. To account for multiple hypothesis testing, the Benjamini-Hochberg (BH) procedure was applied to control the false discovery rate (FDR), and adjusted *p*-values (*p*.adj) are reported. For each significant term, the results presented include the gene ratio, background ratio, and the adjusted *p*-value. Exact *p*-values and the corresponding adjusted *p*-values are provided in the sauce data file. **g** Cell death symptoms induced by transient expression of indicated constructs in *N. benthamiana* and visualized 4 days after infiltration. Red light emission as a result of cell death and its quantitative measurement are shown, alongside the infiltrated leaves. Data are presented as mean ± SD (n1 = 14 leaves, n2 = 5 leaves, n3 = 6 leaves, n4 = 6 leaves, n5 = 12 leaves, n6 = 11 leaves). Statistical analysis was performed using one-way ANOVA followed by Kruskal-Wallis’s multiple comparison tests (*p*.adj < 0.05). Similar results were obtained in three independent experiments.

To directly test their potential genetic interaction, we crossed *siz1-2* with *ka120* and with another autoimmune mutant, *cpr5-1*, generating *siz1-2 ka120* and *siz1-2 cpr5-1* double mutants. We found that *siz1-2 ka120* exhibited a pronounced synergistic phenotype and failed to survive through development (Fig. 1a–c), whereas the *siz1-2 cpr5-1* double mutant displayed a phenotype consistent with additive effects (Fig. 1a, c). These genetic results strengthen a potential functional link between *SIZ1* and *KA120*.

### SIZ1 plays a dual role in plant immune regulation

Since both SIZ1 and KA120 are considered negative regulators of immunity, we further tested their genetic relationship by examining whether overexpressing *SIZ1* could suppress the *ka120* autoimmune phenotype, and vice versa. To this end, we generated both 35S and native promoter-driven *SIZ1* genomic DNA (*SIZ1g*) constructs fused with GFP. The *pSIZ1::SIZ1g-GFP* fusion was functional, as we identified fully complemented lines when the construct was transformed into *siz1-2* plants (Supplementary Fig. 1d). Unexpectedly, we observed an intriguing yet perplexing autoimmune-like phenotype in most *pSIZ1::SIZ1g-GFP* transgenic lines, especially in the wild-type (WT) background, characterized by dwarfism and widespread cell death (Fig. 1d). This phenotype contradicts SIZ1’s previously described role as a negative immune regulator.

The autoimmune phenotype in *pSIZ1::SIZ1g-GFP* is unlikely to result from transgene-induced silencing, as the transgenic plants exhibited strong GFP fluorescence and nuclear accumulation. Phenotypic severity varied among T1 plants: ~34% failed to survive (Category 0), 32% displayed severe dwarfism and extensive cell death in rosette leaves (Category I), 18% developed localized cell death without major growth defect (Category II), and only 16% appeared phenotypically normal (Category III) (Fig. 1d). The phenotype severity appeared to be strongly correlated with *SIZ1* expression levels (Fig. 1e), suggesting that most lines achieved overexpression despite the use of a native promoter. Indeed, most *p35S::SIZ1g-GFP* transgenic plants show a similar autoimmune phenotype (Supplementary Fig. 1e). RNA-seq analysis using *pSIZ1::SIZ1g-GFP* plants with phenotypes showed significant upregulation of genes enriched in defense responses and cell death pathways (Fig. 1f, Supplementary Fig. 1f, and Supplementary Data 1).

Consistent with the observation in *Arabidopsis*, transient expression of *SIZ1g-GFP* in *Nicotiana benthamiana* induces clear onset of cell death (Fig. 1g). Expression of *SIZ1* cDNA (*SIZ1c*) fused to GFP or mCherry also triggers cell death, although less strongly. In addition, we generated a dexamethasone-inducible promoter-driven *SIZ1g-GFP* construct and expressed it in *N. benthamiana* leaves. Three days after dexamethasone treatment, we observed robust cell death response, which did not occur in the mock-treated leaves (Supplementary Fig. 1g).

To determine whether this phenotype directly reflects SIZ1’s catalytic activity, we mutated SIZ1’s catalytic cysteine (C379S) to generate an E3 ligase-dead variant. Neither SIZ1c^C379S^ nor SIZ1g^C379S^ induced cell death when transiently expressed (Fig. 1g). Similarly, among 159 transgenic lines expressing *pSIZ1::SIZ1g*^*C379S*^*-GFP*, only two showed minor growth reduction, and none exhibited cell death even with high expression (Fig. 1e). These results indicate that SIZ1 overexpression phenotype depends on its SUMO E3 ligase activity and highlight a direct role of SIZ1 in promoting immune and cell death activation.

However, overexpressing *SIZ1* in the *ka120* mutant did not further enhance its autoimmune phenotype (Supplementary Fig. 1h), suggesting that SIZ1’s pro-immune function is already activated or capped in the *ka120* background. Indeed, SIZ1 accumulation appeared considerably higher in *ka120* compared to WT (Supplementary Fig. 1i).

Together, these data reveal an unexpected pro-immune and cell-death activity of SIZ1 that depends on its SUMO E3 ligase function, underscoring the limitations of relying solely on loss-of-function mutants to understand SIZ1’s core function and highlighting the importance of maintaining SIZ1 protein homeostasis. Supporting a positive role in immune induction, expression analysis using available databases revealed that *SIZ1* is upregulated under most biotic stress conditions surveyed (Supplementary Fig. 1j).

### Identification of MAC proteins as shared components of the SIZ1 and KA120 proxiome

To investigate how SIZ1 promotes immune activation, we employed an enzyme-catalyzed proximity-labeling approach to identify its in vivo substrates. We fused SIZ1 with TurboID, an engineered promiscuous biotin ligase, and generated stable transgenic *Arabidopsis* expressing *pSIZ1::SIZ1g-TurboID-HA*. Consistent with previous overexpression results, most of the transgenic lines exhibited autoimmune and cell death phenotypes (Supplementary Fig. 2a). Interestingly, lines lacking visible phenotypes failed to induce efficient biotinylation, suggesting low expression levels or impaired protein function in those lines. We used lines displaying autoimmune phenotypes for proximity labeling.

**Fig. 2:**
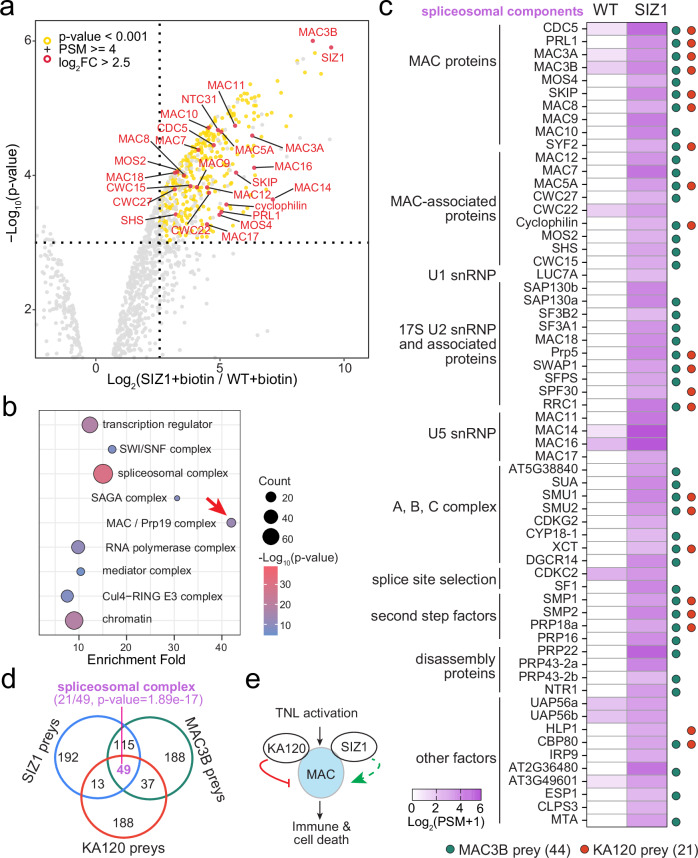
Identification of MAC proteins as shared components of the SIZ1 and KA120 proxiome. **a** Scatter plot showing significantly enriched proteins identified by proximity-labeling proteomics using 10-day-old *pSIZ1::SIZ1g-TurboID-HA* transgenic *Arabidopsis* plants. Biotin-treated WT plants were used as controls for ratiometric analysis. Two biological replicates were used for each sample. SIZ1-specific preys were selected using *p* < 0.001 (two-sided *t*-tests without adjustment), Log_2_fold-change (FC) > 2.5, and peptide spectrum match (PSM) $\ge \,$4 as cutoffs and are represented as yellow and red dots. Identified MAC proteins are labeled in red. See the complete gene list in Supplementary Data 2. **b** GO analysis of SIZ1 proxiome. Representative GO terms and enrichment statistics are shown. Red arrow emphasizes MAC. The statistical significance of enrichment for each GO term was assessed using a hypergeometric test adjusted by the BH procedure, and *p*.adj values are reported. **c** Heatmap showing normalized PSM values of spliceosomal complex proteins identified in the SIZ1 proxiome. Proteins also detected in the MAC3B and KA120 proxiomes are indicated with green and red dots, respectively, on the right. **d** Venn diagram showing overlapping proteins identified in SIZ1, MAC3B, and KA120 proxiome (hypergeometric tests). **e** Schematic diagram depicting potential roles of KA120 and SIZ1 in MAC-dependent immune signaling activation.

Ten-day-old *pSIZ1::SIZ1g-TurboID-HA* and WT plants were treated with free biotin for 5 h to achieve effective proximity labeling (Supplementary Fig. 2b), after which labeled proteins were affinity-purified and identified by label-free quantitative mass spectrometry (LFQMS) with two biological replicates. Proteins significantly enriched in biotin-treated SIZ1-TurboID samples were identified by comparing the peptide spectrum match (PSM) levels against those from biotin-treated WT samples using stringent selection criteria: Log_2_(fold-change) > 2.5, *p*-value < 0.001, and PSM $\ge \,$4 (Fig. 2a). The analysis yielded 369 protein candidates (Supplementary Data 2).

Among the significant candidates, 19 were previously reported as SIZ1 SUMOylation targets^31^, validating the specificity of this dataset (Supplementary Fig. 2c). Gene ontology (GO) analysis revealed that the splicing-related MOS4-Associated Complex (MAC) was the most significantly enriched protein complex (Fig. 2b), with 16 out of 18 previously reported plant MAC components detected (Fig. 2c), missing only MAC13 and MAC15/DHX16^42,43^.

In Arabidopsis, the MAC complex is essential for SNC1-mediated immune signaling^15,43,44^. We previously demonstrated that MAC components are also highly enriched in the KA120 proxiome, where KA120 acts as a chaperone to prevent their spontaneous condensation and maintain their canonical function in splicing. Loss of KA120 and activation of TNL both trigger MAC condensation and the formation of MAC3-dependent nuclear condensates (MDNCs), which are sufficient to activate immune responses^14^. Comparative analysis of the SIZ1 and KA120 proxiomes, along with that of MAC3B—the core MAC scaffold—revealed that the spliceosomal complex, especially MAC components and their associated factors, constitutes the major shared components (Fig. 2c, d). These findings establish a strong molecular and functional association between SIZ1 and KA120, linking both to MAC-dependent immune signaling module (Fig. 2e).

### SIZ1 is recruited by MAC-dependent nuclear condensates to potentiate immune activation and cell death

**Fig. 3:**
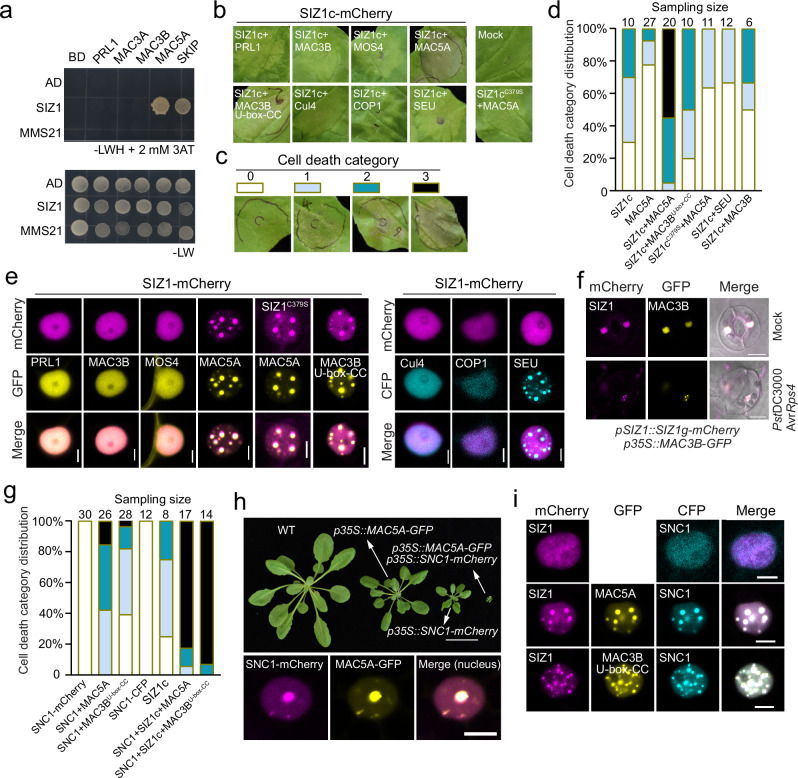
SIZ1 and SNC1 are recruited into MAC nuclear condensates to potentiate immune activation and cell death. **a** Y2H analysis using MAC proteins as the bait and SUMO E3 ligases (SIZ1 and MMS21) as the prey. Zygote yeasts were grown on DDO (SD/-Leu-Trp) and TDO (SD/-Leu-Trp-His) media supplemented with 2 mM 3-AT. Empty vectors were used as negative controls. **b** Cell death triggered by transiently coexpression of SIZ1c-mCherry with GFP-tagged MAC proteins (PRL1, MAC3B, MOS4, MAC5A, MAC3B^U-box-CC^) or CFP-tagged known SIZ1 substrates (Cul4, COP1, SEU) in *N. benthamiana*. Images were taken 4 days after infiltration. Similar results were obtained in three independent experiments. **c** Illustration of cell death categorization: 0 – no symptoms; 1 – chlorosis with localized cell death; 2 – visible cell death in less than 50% of the infiltrated area; 3 – cell death in more than 50% of the infiltrated area. **d**, **g** Distribution of cell death categories in leaves transiently expressing indicated constructs. Data were collected 4 days post-infiltration. Sample sizes are shown above each bar. Similar results were obtained in three independent experiments. **e** Transient expression of mCherry-tagged SIZ1 or SIZ1^C379S^ in combination with GFP-tagged MAC proteins or CFP-tagged SIZ1 substrates in *N. benthamiana*. Pictures were captured 36 h after infiltration. Nuclei were imaged. Scale bars, 5 μm. Similar results were obtained in three independent experiments. **f** Induction of SIZ1 condensates and MDNCs in *p35S::MAC3B-GFP / pSIZ1::SIZ1g-mCherry* double transgenic plants infected with the bacterial pathogen *Pseudomonas syringae* DC3000 avirulent strains carrying effector Avr*Rps4* (OD_600_ = 0.02). Nuclei of guard cells of 2-week-old plants were shown. Scale bars, 5 μm. Similar results were obtained in three independent experiments. **h** Four-week-old soil-grown plants of indicated genetic backgrounds (upper panel). Scale bar, 2 cm. Fluorescence imaging showing co-localization of SNC1-mCherry with MAC5A-GFP condensates in cotyledon cells of the plant (lower panel). A representative nucleus is shown. Scale bar, 5 μm. Similar results were obtained in two independent experiments. **i** Transient coexpression of SIZ1c-mCherry and SNC1-CFP with either MAC5A-GFP or MAC3B^U-box-CC^-GFP in *N. benthamiana*. Nuclei were imaged. Scale bars, 5 μm. Similar results were obtained in three independent experiments.

To investigate how SIZ1 regulates immune and cell death activation through MAC-dependent immune signaling, we first tested its physical association with MAC proteins using Y2H assays. Our results confirmed direct interactions of SIZ1 with at least two MAC proteins, MAC5A and SKIP (Fig. 3a). MMS21, another plant SUMO E3 ligase, did not interact with MAC proteins. We then generated GFP-tagged constructs of MAC proteins (PRL1, MAC3B, MAC5A, and MOS4) and CFP-tagged constructs of a few known SIZ1 substrates (Cul4, COP1, and SEU) for transient expression in *N. benthamiana*. When expressed individually, none of the constructs triggered cell death (Supplementary Fig. 3a). However, co-expression of SIZ1c-mCherry with MAC5A-GFP markedly enhanced cell death, both in frequency (Fig. 3b–d) and intensity (Supplementary Fig. 3b), an effect not observed with SIZ1 cDNA alone or when co-expressed with other SIZ1 substrates. We did not use the SIZ1 gDNA construct for coexpression because the strong cell death it induced masked the enhancement effect.

Fluorescence imaging revealed that MAC5A forms nuclear condensates (Supplementary Fig. 3a), and recruits SIZ1 when coexpressed, prior to the onset of visible cell death (Fig. 3e). In contrast, despite forming spontaneous nuclear condensates, SEU neither recruited SIZ1 (Fig. 3e) nor enhanced SIZ1-mediated cell death when co-expressed (Fig. 3d and Supplementary Fig. 3b). To determine whether SIZ1 recruitment to MAC5A condensates is merely a consequence of cell death, we co-expressed MAC5A-GFP with SIZ1^C379S^-mCherry. The mutant SIZ1 was still recruited by MAC5A-GFP into nuclear condensates, yet no cell death occurred (Fig. 3b–e and Supplementary Fig. 3b). These findings indicate robust and specific interaction of SIZ1 with MAC5A in vivo and their ability to undergo co-condensation.

To further strengthen the functional connection between SIZ1 and the MAC, we leveraged MAC3B, the core scaffolding protein of the MAC complex. We previously demonstrated that MAC3B forms the immune-activating nuclear condensates, MDNCs, following infection with *Pseudomonas syringae* pv. *tomato* DC3000 carrying Avr*Rps4*^14^. Truncation of MAC3B (U-box-CC domain) promotes spontaneous MDNC formation^14^. We found that the MAC3B^U-box-CC^ but not the full-length MAC3B recruited SIZ1 into nuclear condensates in *N. benthamiana* and considerably enhanced SIZ1-mediated cell death, though less strongly than with MAC5A (Fig. 3b–e and Supplementary Fig. 3b). Because MAC3B does not directly interact with SIZ1 in Y2H, we hypothesize that SIZ1 recruitment may occur via MAC5A incorporation into MDNCs. Supporting this, MAC5A colocalized with MAC3B^U-box-CC^ in condensates (Supplementary Fig. 3c). Given the extended intrinsic disordered region (IDR) in MAC5A (Supplementary Fig. 3d) and its strong condensation capacity, MAC5A may serve as an additional scaffolding protein alongside MAC3 during MDNCs assembly. In Arabidopsis, most transgenic *pSIZ1::SIZ1g-GFP* lines with high levels of expression and lethal phenotypes exhibit spontaneous SIZ1 condensate formation (Supplementary Fig. 3e), further supporting the idea that recruitment of SIZ1 to MDNCs potentiates cell death.

We next examined whether SIZ1 recruitment to MAC condensates is a dynamic process that can be triggered during pathogen infection. To this end, we generated a double transgenic line by crossing a low-expressing *pSIZ1::SIZ1g-mCherry* line without autoimmune phenotype with a *p35S::MAC3B-GFP* line. In the absence of pathogen infection, both SIZ1 and MAC3B displayed uniform nuclear distribution without condensation. Upon treatment of *Pst*DC3000 Avr*Rps4*, which triggered TNL-dependent ETI responses, MDNC formation was induced as previously reported, with pronounced accumulation observed in guard cells. Importantly, we observed concurrent relocalization of SIZ1 into MDNCs (Fig. 3f), supporting that SIZ1 associates with MAC proteins during ETI activation and incorporated into MDNCs.

### SIZ1 and SNC1 are co-recruited to the MDNC and function synergistically

Because MAC, SIZ1, and KA120 are all involved in TNL-activated immune signaling, and they all confer genetic interactions with the TNL receptor SNC1^15,19,38,43^, we tested whether SNC1 is also recruited to MAC condensates. Co-expression of SNC1-mCherry with either MAC5A-GFP or MAC3B^U-box-CC^ in *N. benthamiana* and *Arabidopsis* protoplasts showed that both MAC5A and MAC3B^U-box-CC^ effectively recruited SNC1 into condensates (Supplementary Fig. 3f, g). Under our experimental conditions, expressing SNC1-mCherry alone in *N. benthamiana* neither induced SNC1 condensation nor triggered visible cell death, whereas co-expression with MAC5A or MAC3B^U-box-CC^ did both (Fig. 3g and Supplementary Fig. 3g). To confirm the results from transient assays, we crossed *p35S::MAC5A-GFP* and *p35S::SNC1-mCherry* transgenic plants. In F2 progenies expressing both transgenes, we observed severe autoimmune and cell death phenotypes, far exceeding either single transgenic line (Fig. 3h). In the double transgenic plants, SNC1 colocalizes with MAC5A in nuclear condensates (Fig. 3h).

The above findings suggest that both SIZ1 and SNC1 are recruited into MAC nuclear condensates for immune activation. In line with this hypothesis, expression of MAC5A or MAC3B^U-box-CC^ with SIZ1 and SNC1 lead to colocalization of the three proteins in the same nuclear condensates (Fig. 3i). Importantly, this triple co-expression produced the most pronounced cell death observed, surpassing all single or double combinations described above (Fig. 3g). Collectively, these results support a model in which the concurrent recruitment of SIZ1 and SNC1 to MAC condensates simulates maximal activation of immune responses during SNC1-mediated ETI activation.

### MAC nuclear condensates are essential for SIZ1-triggered immune activation

**Fig. 4:**
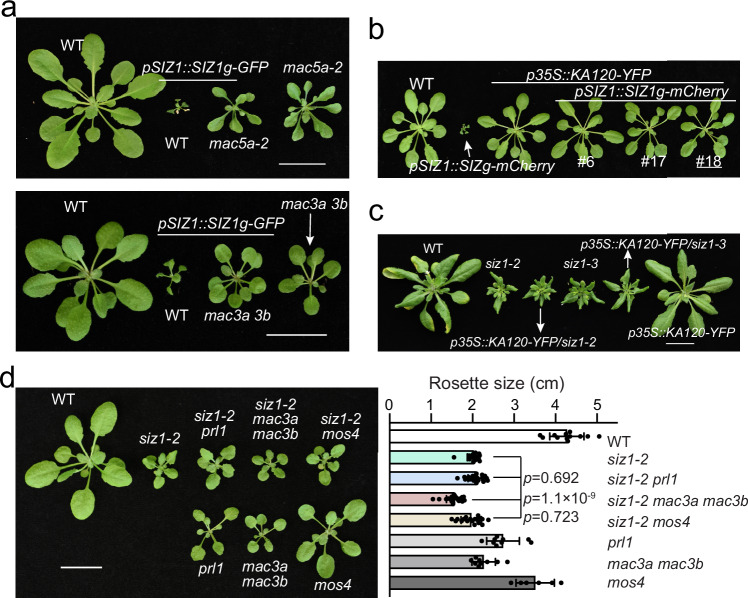
MAC nuclear condensates are essential for SIZ1 overexpression- but not loss-of-function-triggered immune activation. **a**–**c** Four-week-old soil-grown plants of indicated genetic backgrounds are shown. Representative plants are shown. **d** Three-week-old soil-grown plants of indicated genetic backgrounds (left panel) and quantification of the rosette size (right panel). Data are presented as mean ± SD (n_WT_ = 12 plants, n_*siz1-2*_ = 15 plants, n_*siz1-2 prl1*_ = 20 plants, n_*siz1-2 mac3a mac3b*_ = 23 plants, n_*siz1-2 mos4*_ = 18 plants, n_*prl1*_ = 9 plants, n_*mac3a mac3b*_ = 9 plants, n_*mos4*_ = 6 plants). Statistical analysis was performed using one-way ANOVA followed by Dunnett’s multiple comparison tests (adjusted *p* < 0.05). Scale bars, 2 cm. Similar results were obtained in two independent experiments.

To validate the hypothesis that the MDNC functions downstream of SIZ1 and are required for SIZ1-mediated immune and cell death activation, we crossed a *pSIZ1::SIZ1g-GFP* line exhibiting autoimmune and cell death phenotype with the *mac5a* single mutant and the *mac3a mac3b* double mutant. Remarkably, in both mutant backgrounds, the autoimmune phenotype was almost completely suppressed (Fig. 4a and Supplementary Fig. 4a).

We previously showed KA120 overexpression can inhibit MAC condensation and thereby prevent autoimmunity. To further assess whether the condensate, rather than the diffuse form of MAC proteins, is essential for SIZ1-mediated immune activation, we examined whether KA120 overexpression could suppress SIZ1-triggered autoimmune phenotype. Remarkably, when SIZ1 was overexpressed in the *p35S::KA120-YFP* background, no autoimmune phenotype was observed across more than 30 independent transgenic lines with various levels of *SIZ1* expression (Fig. 4b). These results support the conclusion that MAC condensation underlies SIZ1-mediated immune activation. By contrast, overexpressing KA120 did not to rescue the autoimmune phenotype of the *siz1-2* and *siz1-3* mutants (Fig. 4c), indicating that loss-of-SIZ1-triggered immune activation is mechanistically distinct from SIZ1-MAC signaling pathway described here. Consistent with this, MAC mutants also failed to rescue the *siz1-2* mutant phenotype (Fig. 4d). Thus, immune activation in the *siz1* mutant, as well as its previously observed genetic interaction with the *ka120* mutant, is likely to be MAC-independent and potentially involves other substates of SIZ1 and KA120.

Because SNC1 is also recruited to the MDNC to potentiate immune and cell death, we tested its functional contribution to SIZ1-mediated autoimmunity. We crossed *SIZ1g-GFP* line displaying cell death phenotype with the loss-of-function *snc1-r1* mutant. We found that the SIZ1-dependent autoimmune phenotype was largely rescued in the *snc1-r1* mutant (Supplementary Fig. 4b), suggesting that SNC1 is required for SIZ1-MAC-mediated immune induction.

### SIZ1 promotes SUMOylation and stabilization of MAC proteins to sustain the MDNC

**Fig. 5:**
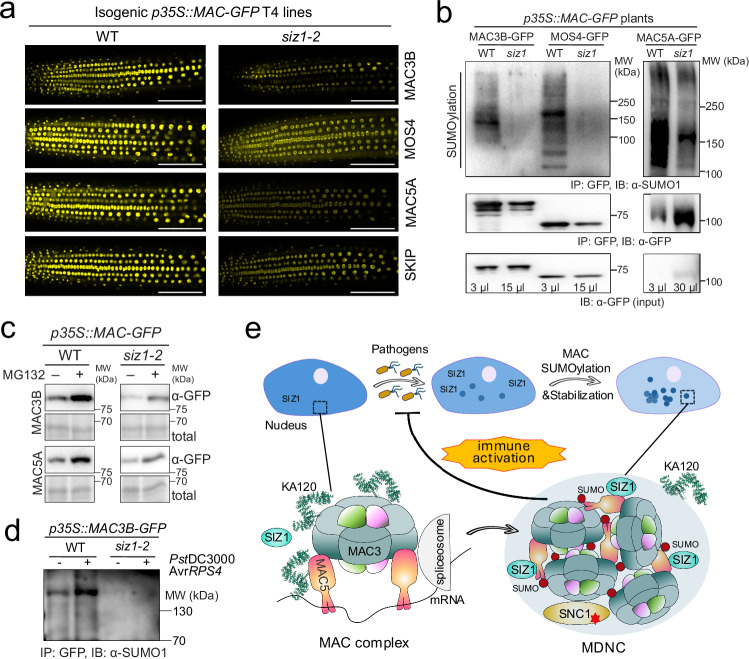
SIZ1 promotes SUMOylation and stabilization of MAC proteins. **a** Subcellular localization of MAC proteins in root cells of 7-day-old WT and *siz1-2* plants. 35S promoter-driven *MAC-GFP* constructs were transformed into *siz1-2* heterozygous plants, and isogenic WT and *siz1-2* lines were obtained for imaging in the T4 generation. Scale bars, 100 μm. Similar results were obtained in five independent transgenic lines. **b** In vivo SUMOylation assay of MAC proteins. Total protein was extracted from 10-day-old isogenic *p35S::MAC-GFP* seedlings treated with 37 °C for 90 min and incubated with GFP-Trap beads. IP samples were immunoblotted with anti-SUMO1 and anti-GFP antibodies. Vertical line indicates SUMOylated MAC proteins. The experiments were performed three times with similar results. The volume of input samples is indicated. **c** In vivo protein stabilization assay. Proteins were extracted from 10-day-old isogenic *p35S::MAC-GFP* seedlings treated with or without the proteasome inhibitor MG132 for 8 h. Extracts were separated by SDS–PAGE and analyzed by immunoblotting with an anti-GFP antibody. Similar results were obtained in three independent experiments. **d** Pathogen-induced SUMOylation of MAC3B. Total protein was extracted from 17-day-old isogenic *p35S::MAC3B-GFP* seedlings infected with the bacterial pathogen *Pst* DC3000 avirulent strains carrying effector Avr*Rps4* (OD_600_ = 0.02) under 28 °C for 24 h and incubated with GFP-Trap beads. IP samples were immunoblotted with anti-SUMO1 antibody. Similar results were obtained in two independent experiments. **e** Working model of SIZ1-mediated regulation of MAC proteins in activating immune signaling.

We next investigated how SIZ1 contributes to immune activation via its interaction with MAC proteins. As a SUMO E3 ligase, SIZ1 can modulate the activity, localization, or stability of its substrates through SUMOylation. To test whether MAC proteins are SUMOylated in vivo and whether this requires SIZ1, we transformed GFP fusion constructions of MACs, including MAC3B, MOS4, MAC5A, and SKIP, into the *siz1-2* heterozygous background. We selected T1 transformants heterozygous for *siz1-2* and subsequently obtained isogenic *MAC-GFP* lines in WT and *siz1-2* backgrounds. We did not observe any localization change of these MAC proteins in *siz1-2* compared to WT (Fig. 5a).

Most MAC proteins possess predicted SUMOylation sites or have been experimentally identified as SUMOylated in proteomic studies (Supplementary Fig. 5a). Consistent with this, immunoblot analysis using an anti-SUMO1 antibody revealed in vivo SUMOylation of MAC3B, MOS4, and MAC5A in WT plants (Fig. 5b). These SUMOylation events were most evident upon heat treatment (e.g., 28 ~ 37 °C), as basal SUMOylation levels under 22 °C were minimal. SUMOylation of MAC proteins was almost completely abolished in the *siz1-2* mutant (Fig. 5b), indicating that their SUMOylation depends on SIZ1. Moreover, we found that SIZ1 exhibited physical interaction with MAC5A in yeast (Fig. 3a), suggesting MAC proteins may be direct substrates of SIZ1. Although not all MAC interacted with SIZ1 in Y2H assays, we detected strong bimolecular fluorescence complementation (BiFC) signal between SIZ1 and all tested MAC proteins in the nucleus (Supplementary Fig. 5b). Many of these interactions were observed to concentrate within nuclear condensates. These results suggest that SIZ1 promotes SUMOylation of MAC proteins upon association with the MAC complex.

In these analyses, we consistently observed reduced accumulation of MAC proteins in the *siz1-2* mutant (Fig. 5a, b). To substantiate this observation, we performed quantitative analyses of MAC protein levels using isogeneic *MAC-GFP* transgenic lines in WT and *siz1-2* backgrounds. We found that the abundance of MAC proteins significantly increased upon MG132 treatment, but this did not occur in the *siz1-2* background (Fig. 5c). This enhanced MAC accumulation in WT is likely due to reduced proteasomal degradation, further supported by the cycloheximide chase experiment (Supplementary Fig. 5c). These results suggest that MAC protein undergo continuous turnover and that SIZ1 plays a negative role in this process. Consistently, polyubiquitination of MAC5A was considerably elevated in the *siz1-2* mutant (Supplementary Fig. 5d). These findings support that MAC proteins are in vivo targets of SIZ1 and indicate a correlation between the loss of SUMOylation and reduced protein stability for MAC in the absence of SIZ1.

To test if MAC SUMOylation is affected by immune induction, we inoculated *MAC3B-GFP* transgenic plants with *Pst*DC3000/Avr*Rps4* under 28 °C. We found that SUMOylation of MAC3B is significantly increased compared mock treatment (Fig. 5d). We propose that SIZ1 plays a role in promoting or sustaining MDNC stability by SUMOylating MAC proteins following the initiation of MAC condensation upon immune induction.

### A working model for SIZ1-dependent immune activation via promoting/sustaining MDNCs

Our results indicate a model in which pathogen infection induces SIZ1 upregulation, leading to enhanced SUMOylation of MAC proteins and increased protein stability (Fig. 5e). As both elevated protein abundance and SUMOylation modification are established drivers of protein condensation^45,46^, SUMOylation and accumulation of MAC proteins likely increase their condensation propensity and maintain their high local concentrations within MDNCs. This, in turn, stabilizes the condensate scaffold and supports MDNC-mediated immune signaling. Consistent with the model, mutation of four lysine residues (K to R) predicted to be SUMOylated in MAC5A abolished its SUMOylation and ability to form nuclear condensates (Supplementary Fig. 5e, f). Moreover, fluorescence recovery after photobleaching (FRAP) analysis showed that coexpression of SIZ1 markedly reduced the exchange rate of MDNC-localized MAC5A with the surrounding nucleoplasmic pool (Supplementary Fig. 5g), suggesting that SUMOylated MAC is more stably incorporated or interlocked within the condensates.

The above mechanism appears to operate antagonistically to the KA120-dependent inhibitory pathway that prevents MDNC formation under homeostatic conditions. These complementary regulatory pathways likely function under distinct cellular states to coordinate MDNC dynamics and control immune activation. We speculate that SIZ1 recruitment to MDNCs may also sequester it from other nuclear substrates, functionally recapitulating aspects of the *siz1* mutant phenotype and further amplifying immune responses through MDNC-independent mechanisms.

## Discussion

Although *siz1* mutants exhibit autoimmunity, which led to the original designation of SIZ1 as a negative immune regulator, this phenotype likely reflects the widespread loss of SIZ1-mediated SUMOylation across diverse substrates. Thus, using loss-of-function *siz1* mutants is insufficient to define SIZ1 function across multiple levels. In contrast, the pro-immune activity of SIZ1 specifically operates through its interaction with MAC and recruitment to MDNCs, where it SUMOylates and stabilizes MAC proteins to potentiate MDNC-based immune and cell death activation. This reveals one precise immune function of SIZ1.

Consistent with this distinction, the immune pathways engaged by SIZ1 overaccumulation and by SIZ1 loss-of-function appear different. Loss of MAC or KA120 overexpression, which disassembles MDNCs, effectively rescues the SIZ1 overexpression phenotype but fails to suppress *siz1-2* mutant autoimmunity (Fig. 4). Moreover, *siz1-2* mutant does not form MDNCs despite robust immune activation (Fig. 5a), indicating that *siz1-2* autoimmune phenotype is MDNC-independent. Nevertheless, sequestration of SIZ1 into MDNCs during pathogen challenge may mimic aspects of *siz1-2* loss-of-function by limiting SIZ1 availability for other nuclear substrates, thereby promoting MDNC-independent immune activation.

SIZ1 overaccumulation is physiologically relevant, as its expression is broadly induced by biotic stress (Supplementary Fig. 1j), and its recruitment to MDNCs can be triggered by *Pst*DC3000/Avr*Rps4* infection (Fig. 3f). SIZ1-promoted MDNC formation and KA120-dependent MDNC inhibition represent complementary mechanisms that coordinate pro-immune condensate dynamics to launch defense, illustrating a system that balances condensate assembly and disassembly to prevent autoimmunity while preserving the capacity for rapid defense responses.

The recruitment of both SIZ1 and SNC1 to MAC condensates, along with the resulting enhancement of cell death activity, exemplifies a convergent cellular hub for immune activation. Although not yet observed under pathogen infection conditions, this suggests that TNL condensates and MDNCs may represent partially overlapping condensate populations that are central to nuclear immune signaling.

Several important questions remain for future investigation. Our current data do not provide direct evidence that MAC proteins are more SUMOylated in the condensed form than in the diffuse form, owing to the technical difficulty of isolating pure MDNCs, although both may result in the reinforcement of MDNCs. Moreover, the predicted SUMOylation sites may also be subject to ubiquitination, and in this context the two modifications may exert opposing effects on MAC protein stability. This duality complicates interpretation of results obtained using MAC^KR^ mutants and highlights the potential interplay between SUMOylation- and ubiquitination-dependent regulation of MAC activity, which warrants further investigation. The contextual nature of SIZ1’s immune function also raises intriguing questions about how plants balance immune activation with other stress responses, given the wide range of stress and signaling-related substrates targeted by SIZ1. While our studies establish SIZ1’s pro-immune capacity, understanding the exact physiological conditions that transiently elevate SIZ1 activity or enhance its recruitment to MAC condensates, without triggering unrelated cellular responses, will be crucial for fully appreciating this regulatory mechanism.

## Methods

### Plant materials and growth conditions

All *Arabidopsis* plants used in this study are in the Columbia-0 (Col-0) background. Arabidopsis were grown either on Sunshine Mix#4 soil or on ½ MS medium (Caisson Labs) with 1% agar and 1% sucrose (pH 5.7) under a light cycle 12 h light/12 h dark at 22 °C. *N. benthamiana* were grown under a 16 h light/8 h dark–light cycle at 22 °C. Seeds were surfaced-sterilized with 10% (v/v) bleach supplemented with 0.01% Triton-X for 5 min and washed thoroughly in sterile water 6 times before germinating on MS medium. The *ka120* (Salk_148803), *siz1-2* (Salk_065397), *siz1-3* (Salk_034008), *prl1-2* (Salk_039427), *mac3a mac3b* (Salk_089300, Salk_050811), *mos4-1* (CS69914), *mac5a-1* (Salk_132881), *mac5a-2* (Salk_142085), and *abi5* (Salk_013163) mutants were obtained from Arabidopsis Biological Resource Center (ABRC). The *snc1-r1* and *cpr5-1* were obtained from Yuelin Zhang lab and Xinnian Dong lab, respectively. All higher order mutants in this study were obtained through genetic crosses. All primers for mutant genotyping are listed in Supplementary Data 3.

### Microbe strains

*Escherichia coli* strain DH5α was grown at 37 °C in LB medium with antibiotics, the resistance to which is conferred by the plasmid. *Agrobacterium tumefaciens* strain GV3101 was grown at 28 °C in LB medium with antibiotics, the resistance to which is conferred by the plasmid. *Pseudomonas syringae* pv. tomato strain DC3000 carrying effector Avr*Rps4* was grown at 28 °C in a modified LB medium with antibiotics as described. *Saccharomyces cerevisiae strain* Y187 and AH109 were grown at 30 °C in YPDA medium or synthetic dropout medium as described.

### Plasmid construction and transgenic plant generation

To generate fluorescence protein-tagged binary constructs, the full-length coding sequence of *SIZ1* (AT5G60410.2) / *Cul4* / *MAC3B*^*U-box-CC*^ and the full-length genomic DNA of *SIZ1* / *SNC1* / *MAC3B* / *MOS4* / *MAC5A* / *SKIP* / *SEUSS* / *COP1* were inserted into modified binary vector pCambia1300 with the GFP tag or mCherry tag and pEarleyGate100 with CFP tag at the C terminus by In-Fusion cloning (ClonExpress II One Step Cloning Kit, Vazyme). The *SIZ1* native promoter was amplified together with its genomic sequence and cloned into the expression cassette of modified vector pCambia1300-GFP, pCambia1300-TurboID-HA, and pBI111L-mCherry without 35S promoter by In-Fusion cloning. *SIZ*^*C379S*^ were generated by site-directed mutagenesis with the primers listed in Supplementary Data 3. To generate the Dex-inducible *SIZ1-GFP*, full-length genomic DNA of *SIZ1* was cloned into the entry vector pBSDONR p1-p4, and GFP tag was cloned into pBSDONR p4r-p2 using the BP reaction (BP Clonase II, Invitrogen). The resulting pBSDONR p1-p4 and pBSDONR p4r-p2 constructs were combined and cloned into the destination vector pBAV154 with a Dex-inducible promoter using the LR reaction (LR Clonase II, Invitrogen). All destination vectors were transformed into WT, *siz1-2* heterozygous plants, *ka120* heterozygous plants or *35S:KA120*-*YFP* homozygous plants using *Agrobacterium tumefaciens* GV3101-mediated floral dipping. Glufosinate-ammonium/Basta (25 μg/ml, Supelco), kanamycin A (50 μg/ml, Gold Biotechnology), or hygromycin B (25 μg/ml, Invitrogen) was used for screening transgenic plants. For BiFC transient assays in *Arabidopsis* protoplasts, the full-length genomic DNA of *SIZ1* and *PRL1* / *MAC3A* / *MAC3B* / *MOS4* / *MAC5A* / *SKIP* were cloned into *pUC18-p35S::nYFP* and *pUC18-p35S::cYFP* by In-Fusion cloning. For in vivo SUMOylation assay in protoplasts, the coding sequence of SUMO1 was inserted into pHBT95-2xFLAG vector using In-Fusion Cloning. To create constructs for Y2H assays, the coding sequence of *SIZ1* and *MMS21* (AT3G15150.1) were inserted into the pGADT7 vector using In-Fusion Cloning. pGBKT7-*PRL1* / *MAC3A* / *MAC3B* / *MAC5A* / *SKIP* were described previously. All PCR products were obtained using high-fidelity DNA polymerase (Phanta Max, Vazyme). All primers for cloning are listed in Supplementary Data 3.

### RNA extraction and RT-qPCR

For RT-qPCR, total RNA was extracted from plant tissues using TRIzol reagent (Invitrogen). First-strand cDNA was synthesized from 1 μg of total RNA using HiScript III 1st Strand cDNA Synthesis Kit (Vazyme, R312). qPCR was performed using Taq Pro Universal SYBR qPCR Master Mix (Vazyme, Q712). Primers used for qPCR are provided in Supplementary Data 3. *ACTIN2* (AT3G18780) was used as the reference gene.

### RNA-seq and analysis

For RNA-seq, rosette leaves from four-week-old WT and *pSIZ1:SIZ1g-GFP* transgenic plants were collected, and total RNA was extracted using the Direct-zol RNA Miniprep kit (Zymol Research). Library construction, quality control, and RNA sequencing were performed by Novogene (https://en.novogene.com/) on an Illumina NovaSeq X Plus Series (PE150) platform. The raw RNA-seq reads generated were filtered using trim-galore v0.6.6 with the default parameters to remove low-quality reads. Cleaned data were then mapped to the *Arabidopsis thaliana* genome TAIR10 using HISAT2 v2.2.051 with default parameters. BAMs were sorted by Samtools v1.9.52. Read counts of each gene were generated using HTseq-count with the default parameters. Differences in gene expression were analyzed using the DESeq2 package (v.3.20, https://bioconductor.org/packages/release/bioc/html/DESeq2.html) in R. Differentially expressed genes were determined under the criteria of adjusted *p*-value < 0.05 and fold change > 2.

### Proximity labeling and affinity purification

TurboID-based proximity labeling and affinity purification of biotinylated protein were performed as described previously with minor modifications^47^. In brief, ten-day-old *pSIZ1::SIZ1g-TurboID-HA* transgenic seedlings and WT non-transformants were treated with 50 μM free biotin for 5 h at room temperature. Two biological replicates were used per sample. For each replicate, 0.5 g of biotin-treated seedlings were harvested and frozen in liquid nitrogen. The material was ground into a fine powder, and total protein was extracted using 2.5 ml of protein extraction buffer (50 mM Tris, pH 7.5, 150 mM NaCl, 0.5% Triton X-100, 0.5% Nonidet P-40, 0.5% sodium deoxycholate, protease inhibitor cocktail (Roche), and 40 μM MG132 (Sigma-Aldrich). Protein extracts were clarified by centrifugation at 16,000 × g for 15 minutes at 4 °C. Free biotin was removed from biotin-treated samples using PD-10 desalting columns (Cytiva) according to the manufacturer’s instructions. For affinity purification, streptavidin-coated magnetic beads (Dynabeads MyOne Streptavidin C1, Invitrogen) were incubated with desalted protein extracts overnight at 4 °C with gentle rotations. Beads were then washed five times with wash buffer (50 mM Tris, pH 7.5, 150 mM NaCl, 0.5% Triton X-100, 0.5% Nonidet P-40, 0.5% sodium deoxycholate) and one time with 1 M cold KCl, with each wash consisting of 5 min incubations at 4 °C followed by magnetic separation.

### On beads digestion, mass spectrometry, and proteomics analysis

For on-beads tryptic digestion, streptavidin beads were washed three times with PBS buffer and incubated overnight at room temperature with gentle shaking in 100 μL of 50 mM triethylammonium bicarbonate buffer (TEAB) containing 1 μg trypsin. The resulting peptides were separated from the beads using a magnetic rack and dried in a Speedvac. The peptides were then resuspended in 10 μL 0.1% trifluoroacetic acid (TFA) and desalted using 10 μL C18 ZipTips according to the manufacturer’s instructions (Thermo Fisher). The purified peptides were subsequently dried and reconstituted in 20 μL formic acid (FA) prior to LC-MS/MS analysis.

LC-MS/MS was performed on a Orbitrap Eclipse Tribrid Mass Spectrometer (Thermo Fisher) coupled to an Easy-NLC 1200 system. Peptides were first trapped an Acclaim PepMap 100 C18 trapping column (75 μm particle size, 2 cm bed length) and separated on an Aurora Series C18 emitter Column (25 cm × 75 µm, 1.7 µm; IonOpticks) at a flow rate of 300 nL min^−^^1^. Peptides were eluted using a gradient from 3 to 28% solvent B (80 % acetonitrile, 0.1 % formic acid) over 106 min, followed by 28–44% solvent B over 15 min and a 15 min wash at 90 % solvent B.

Data dependent acquisition was performed with a precursor scan range of m/z 375–1600 (resolution 120,000; AGC 2 × 10^5^, maximum injection time 50 ms. The most intense multiply charged ions were selected for fragmentation (resolution 15,000, AGC 5 × 10^4^, maximum injection time 22 ms, isolation window 1.4 m/z, charge states 2–8, cycle time 3 s). Fragmentation was performed using higher-energy collision dissociation (HCD) with normalized collision energy of 27 and dynamic exclusion of 30 s.

Raw data were processed using FragPipe (version 22.0). Database searches were performed with MSFragger (version 4.1, https://msfragger.nesvilab.org/) against the Araport11 Arabidopsis thaliana proteome with precursor and fragment mass tolerance of 10 ppm and 20 ppm, respectively. Carbamidomethylation of cysteine was set as a fixed modification, while protein N-terminal acetylation, glutamine to pyroglutamate conversion at the peptide N-terminus, and methionine oxidation were set as variable modifications. Up to one missed cleavage and two variable modifications per peptide were allowed. Quantification was performed using IonQuant (version 1.10.27) with MaxLFQ enabled, a minimum ion count of two, and match-between-runs enabled. Peptide and protein false discovery rates were controlled at 1%. LFQ intensities were log₂-transformed and further processed in Perseus (v2.1.3). Non-*Arabidopsis thaliana* proteins were removed, and proteins identified in fewer than two replicates of any sample group were filtered out. Missing values were imputed from a normal distribution (width = 0.3, downshift = 1.8). Differential enrichment analysis was performed using the DEP R package (v.3.20, https://bioconductor.org/packages/release/bioc/html/DEP.html) in R^48^.

### Transient protein expression and cell death quantification

Agrobacterium-mediated transient protein expression in *N. benthamiana* was performed as previously described in ref. ^49^. For coexpression assays, Agrobacterial solution mixtures were infiltrated using a needless syringe into leaves of four-week-old *N. benthamiana* plants. About 36 h after infiltration, fluorescence images were acquired in leaf epidermal cells using a Zeiss LSM880 confocal microscope equipped with a 63× oil objective lens. Cell death phenotypes were documented at 4 days post-infiltration. The red-light channel signal was imaged and measured as previously reported^50^. Treated plant material was placed in a ChemiDoc MP imaging system (Bio-Rad), and images were acquired on excitation by a light source in the green visible spectrum (Green LED Module kit no. 1708284), with filters capturing the light emitted in the red visible spectrum (filter 605/50). The exposure time was adjusted to avoid image saturation. The mean intensity of red channel signal was measured by Fiji. For Dex-inducible transient *SIZ1* expression assays, 40 µM dex or water was sprayed 16 h after *Agrobacterium* infiltration, and the pictures were captured 3-days post-infiltration.

### Drug and pathogen treatment

For zeocin treatment, WT and mutant seeds were germinated and grown on ½ MS media [1/2Murashige & Skoog Basal Medium (Caisson Labs), 0.05% MES, 1% sucrose, 0.5% Phytagel] with or without 2 µg/ml zeocin (Thermo Scientific). Root length was measured after 7-days growth using Fiji. For ABA treatment, six-day-old seedlings grown on ½ MS media were transplanted into ½ MS media with or without 15 µM ABA (Sigma). Pictures were captured after 13-days growth. For pathogen treatment, *Pst*DC3000/Avr*Rps4* was grown at 28 °C on a modified LB medium containing 25 µg/mL rifampicin and 50 µg/mL kanamycin, and until an OD_600_ of 0.8 was reached. Bacteria were collected by 3000 g centrifugation, washed, and diluted to the OD_600_ = 0.02 with ½ MS liquid media. Two-week-old plate-grown seedlings were transferred into 6-well plate containing 5 mL ½ MS media plus *Pst*DC3000/Avr*Rps4* (OD_600_ = 0.02). Images were captured after 24 h by Zeiss LSM880 equipped with a 63× oil objective lens.

### In vivo SUMOylation and ubiquitination analysis

Ten-day-old transgenic seedlings grown on ½ MS media were incubated in the water at 37 °C for 90 min. 17-day-old transgenic seedlings grown on ½ MS media were infected with the bacterial pathogen *Pseudomonas syringae* DC3000 avirulent strains carrying effector Avr*Rps4* (OD_600_ = 0.02) under 28 °C for 24 h. The samples were dried with paper towels and weighed in proportion to buffer volume for each sample. Total protein from 0.4 g seedlings was extracted with 2 mL protein extraction buffer [150 mM NaCl, 50 mM Tris-HCl (pH 7.5), 1 mM EDTA, 1% (w/v) Triton X-100, 1 mM dithiothreitol (DTT), 20 mM N-ethylmaleimide, and Protease Inhibitor Cocktail (Roche)].

For in vivo SUMOylation assay in protoplasts, 400 ng *p35S::FLAG-SUMO1* plasmid was transfected into 5 mL protoplasts (1 × 10^6^ protoplasts) isolated from *p35S::MAC5A-GFP* or *p35S::MAC5A*^*4KR*^*-GFP* transgenic plants. Following incubation at 22 °C for 16 h, protoplasts were subjected to heat treatment at 37 °C for 30 min prior to protein extraction.

The protein extracts were incubated with GFP-Trap beads (ChromoTek, gta-20) at 4 °C for 3 h. The beads were collected via centrifugation at 2,000 g for 5 min and washed three times with washing buffer [150 mM NaCl, 50 mM Tris-HCl (pH 7.5), 1 mM EDTA, 0.2% Triton X-100, 1 mM DTT]. Input and immunoprecipitated proteins were analyzed via immunoblotting with anti-SUMO1 (1:500 ~ 1:1,000, Abcam, Ab5316), anti-GFP antibodies (1:5,000, Clontech, 632381), anti-FLAG (1:1000, Sigma-Aldrich, F1804) or with anti-ubiquitination (1:1000, Santa Cruz, sc-8017).

### In vivo protein degradation assay

Ten-day-old transgenic seedlings grown on ½ MS media were treated with or without 50 μM MG132 for 8 hours. The samples were dried with paper towels and normalized by fresh weight. Samples were then frozen in liquid nitrogen and ground to fine powder. Protein was extracted by 2 × SDS buffer [125 mM Tris-HCl (pH 6.8), 4% SDS, 20% glycerol] at 95 °C for 5 min. The supernatant was collected after centrifuged at 16,000 g for 10 min. Protein samples were separated by SDS-PAGE, and subject to immunoblotting using anti-GFP (1:5,000, Clontech, 632381) and anti-Actin (1:1000, Abiocode, R3772-1P). The total protein visualized by stain-free assay was used as loading control.

### Yeast two hybrid

Y2H analysis was performed as reported in ref. ^51^. The activation domain fusion (prey) constructs and the DNA-binding domain fusion (bait) constructs were transformed into yeast strains Y187 and AH109, respectively. The yeast cells of Y187 and AH109 containing corresponding constructs were mated in 2 × YPDA medium at 30 °C for 24 h before being plated. Diploid yeasts were grown on double (SD-Leu-Trp) or triple dropout (SD-Leu-Trp-His) media containing 2 mM 3-amino-1, 2, 4-Trizole (3-AT) at 30 °C for 5–7 days.

### Protoplast transfection

Plasmids used for protoplast transfection were purified using NucleoBond Xtra Midi EF (Takara Bio). Arabidopsis mesophyll protoplasts were prepared and transfected as previously described in ref. ^52^. Briefly, one-month-old Arabidopsis leaves were sliced and digested in enzyme lysis solution [0.4 M mannitol, 20 mM KCl, 20 mM MES (pH 5.7)] containing 1.5% cellulase R-10, 0.4% macerozyme R-10 (Yakult pharmaceutical IND. CO), 10 mM CaCl_2_, 5 mM β-mercaptoethanol and 0.1% BSA for 3 hours to produce protoplasts. For BiFC co-transfections, 10 µg of each plasmid was used to transfect 200 µl protoplasts (4 × 10^4^ protoplasts). After transfection, protoplasts were incubated for 12–16 h for other assays.

### FRAP analysis

FRAP of MAC5A-GFP condensates in *N. benthamiana* leaves was performed on a Zeiss LSM900 using the 40x oil objective. MAC5A condensates were bleached using a laser intensity of 100% at 488 nm with 30 iterations. Fluorescence recovery was recorded for 1 min with 3 s intervals after bleaching. Analyses of the mean fluorescence intensity of the bleached region were carried out using Image J and the recovery curve was drawn using GraphPad Prim 10.

### Reporting summary

Further information on research design is available in the Nature Portfolio Reporting Summary linked to this article.

## Supplementary information

Supplementary Information

Descriptions of Additional Supplementary Files

Supplementary Data 1

Supplementary Data 2

Supplementary Data 3

Reporting Summary

Transparent Peer Review file

## Source data

Source Data

## Data Availability

Raw data files for the RNA-seq data generated in this study have been deposited into the NCBI GEO under accession number GSE307237 and are publicly available. Raw data files for all mass spectrometry analyses generated in this study have been deposited to the ProteomeXchange Consortium via the PRIDE partner repository under accession number PXD067497 and are publicly available. Source data are provided with this paper. The RNA-seq and proteomics data generated in this study are provided in the Supplementary Data 1 and 2, respectively. Source data are provided with this paper.
