# Supplementary material for: SUMO E3 ligase SIZ1 promotes nuclear condensate-mediated immune activation in Arabidopsis
Source: Nat Commun. 2026 Apr 15;17:5248. doi: 10.1038/s41467-026-72063-x (PMC13260931; doi:10.1038/s41467-026-72063-x)
Supplement: Supplementary file 1 — Supplementary Information [file 41467_2026_72063_MOESM1_ESM.pdf]

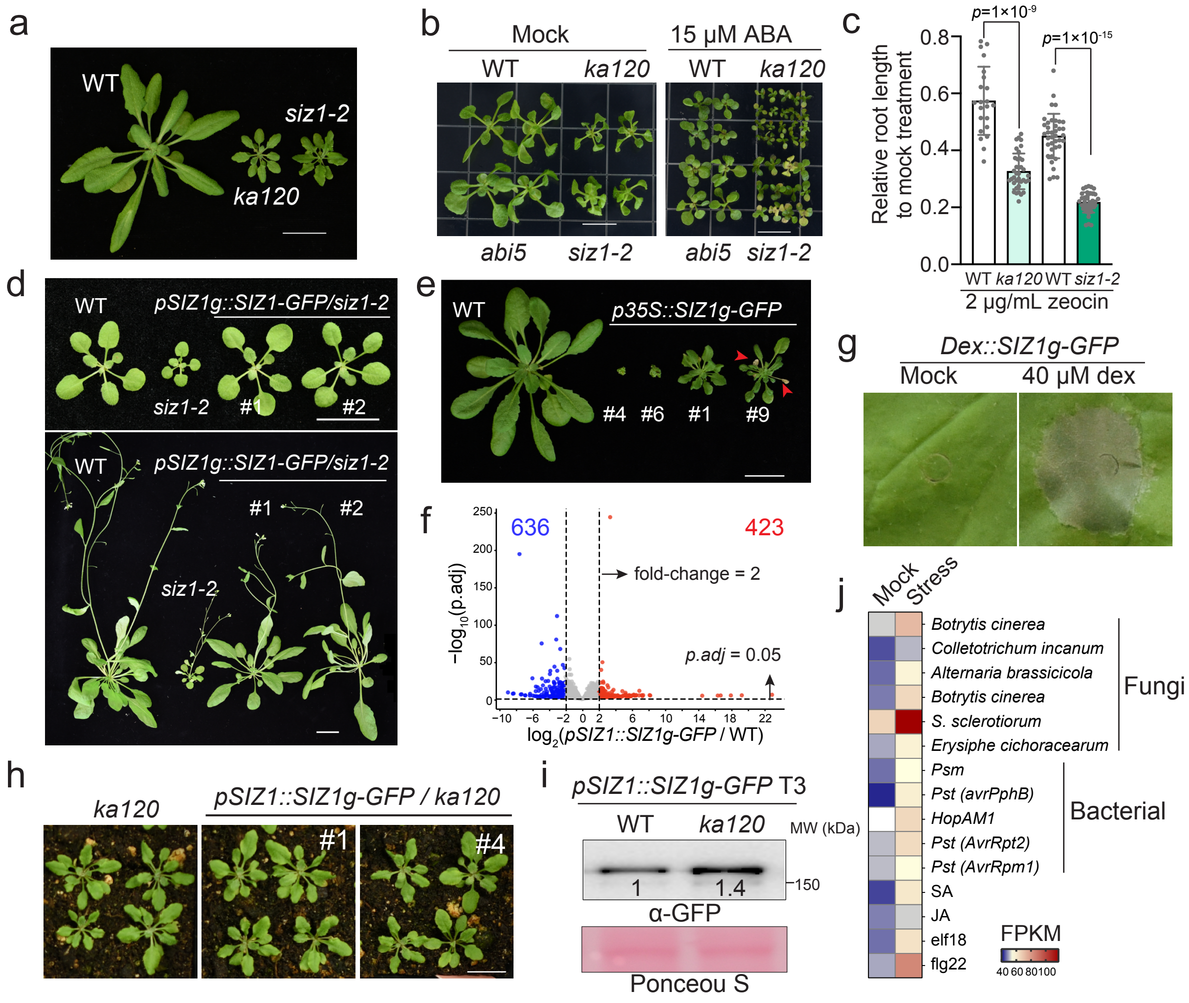

Supplementary Figure 1. Overexpression of SIZ1 promotes immune and cell death activation

(**a**) Five-week-old soil-grown WT, *ka120,* and *siz1-2* mutant plants. Scale bar, 2 cm. Similar results were obtained in three independent experiments.

(**b**) Six-day-old WT, *ka120, abi5*, and *siz1-2* seedlings were transferred onto medium supplemented with or without 15 μM ABA. Pictures were captured after a 13-days growth. Scale bars, 1 cm. Similar results were obtained in three independent experiments.

(**c**) Measurement of plant response to DNA damage inducing reagent zeocin. Plants were grown on 1/2 MS media supplied with or without 2 μg/mL zeocin for 7 days. The root length of zeocin-treated samples was normalized to mock-treated samples. Data are presented as mean ± SD (n = 22 and 37 for WT and *ka120*, n = 39 for WT and *siz1-2*, two-tailed Welch's t-test, $p < 0.01$). Similar results were obtained in three independent experiments.

(**d**) Three-week-old (top) and seven-week-old (bottom) soil-grown WT, *siz1-2,* and indenpendent T2 transgenic lines of *pSIZ1::SIZ1g-GFP / siz1-2*. Scale bars, 2 cm. Similar results were obtained in three experiments.

(**e**) Six-week-old soil-grown WT and independent *p35S::SIZ1g-GFP* transgenic lines. Scale bar, 2 cm. Similar results were obtained in three independent experiments.

(**f**) Volcano plot showing differentially expressed genes (DEGs) (p.adj < 0.05 and fold change >2, two-tailed t tests with Benjamini-Hochberg adjustment) in *pSIZ::SIZ1g-GFP* compared with WT determined by RNA-Seq. Up- and down-regulated DEGs are displayed as red and blue dots, respectively. The rosette leaves of 4-week-old plants were sampled, and two biological replicates were used for RNA-seq. See Supplementary Data 1 for the DEG list.

(**g**) Agrobacterium carrying *Dex::SIZ1g-GFP* construct was infiltrated into *N. benthamiana* leaves and incubated for 16 h before 40 μM dex treatment. Images were captured 3 days post-treatment. Similar results were obtained in three independent experiments.

(**h**) Four-week-old soil-grown *ka120* plants and indenpendent *pSIZ1::SIZ1g-GFP / ka120* transgenic lines. Scale bar, 1 cm. Similar results were obtained in three independent experiments.

(**i**) Protein from T3 isogenic *pSIZ1::SIZ1g-GFP* in the WT and *ka120* mutant seedlings was extracted and separated on SDS–PAGE gel followed by immunoblotting with anti-GFP antibody. Relative band intensities of SIZ1-GFP were quantified by Fiji. The value of samples from *pSIZ1::SIZ1g-GFP* / WT was set as 1. Similar results were obtained in three independent experiments.

(**j**) Heatmap of expression levels of *SIZ1* upon inoculation with different pathogens or immune elicitors compared to mock treatment. FPKM: fragments per kilobase of transcript per million mapped reads. Data source: Arabidopsis RNA-seq Database (https://plantrnadb.com/athrdb/).

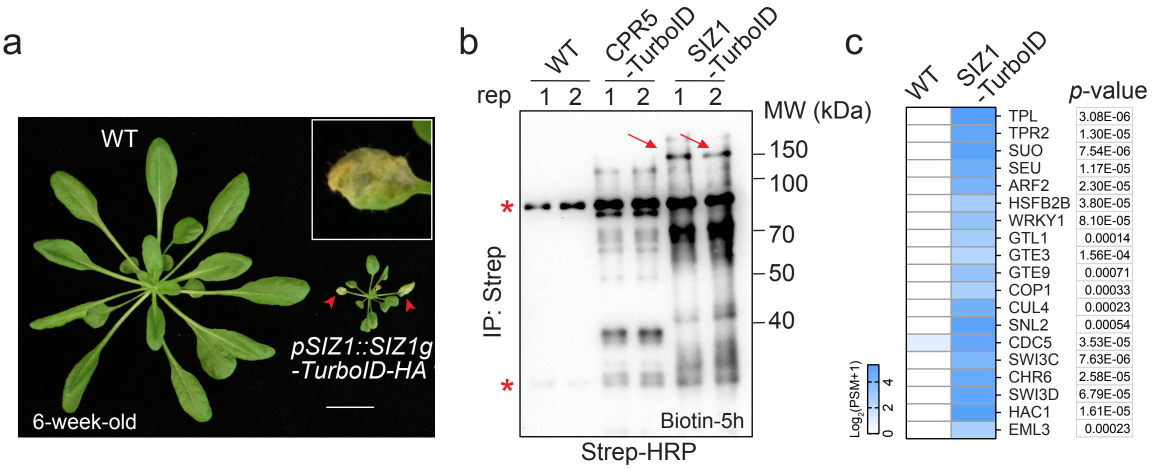

Supplementary Figure 2. Profiling of SIZ1 proxiome

(**a**) Six-week-old soil-grown WT plant and a representative *pSIZ1::SIZ1g-TurboID-HA* transgenic line. Scale bar, 2 cm. Similar results were obtained in three independent experiments.

(**b**) Inducible biotinylation of protein in the *SIZ1-TurboID*, *CPR5-TurboID*, and WT non-transgenic plants. Transgenic and WT seedlings were treated with 50 μM free biotin for 5 hours before total protein was extracted. Samples were subjected to affinity purification by streptavidin-conjugated beads. About 5% sample was aliquoted for immunoblotting using HRP-conjugated streptavidin antibody. Arrows indicate self-biotinylated SIZ1-TurboID-HA protein, and asterisks indicate naturally biotinylated proteins.

(**c**) Heatmap displaying normalized PSM values of previously reported SIZ1 substrates identified by SIZ1-TurboID proximity labeling. Statistics of MS results for each protein are shown on the right.

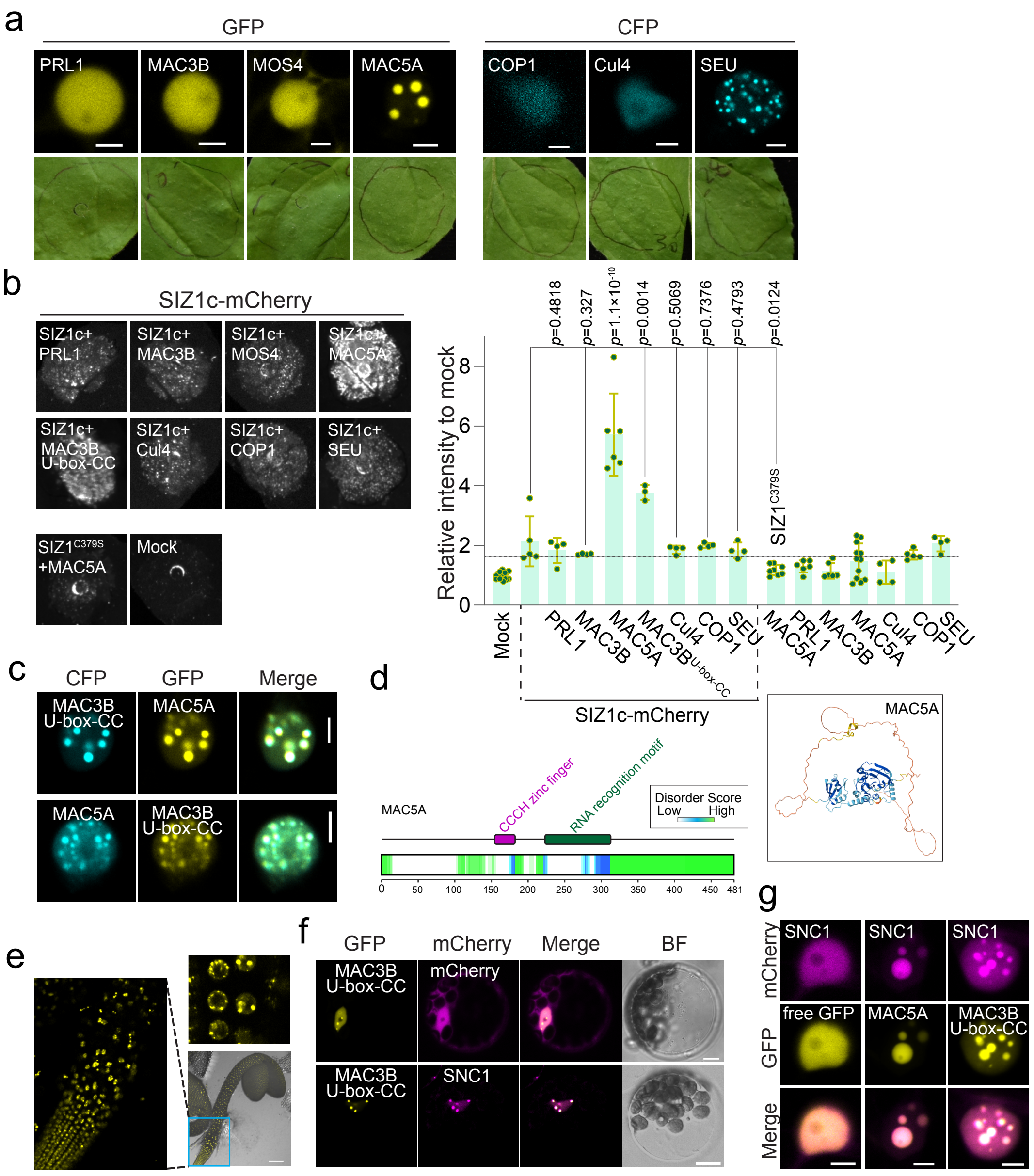

Supplementary Figure 3. SIZ1 and SNC1 are recruited into MAC nuclear condensates to trigger cell death
(**a**) Transient expression of individual GFP-tagged MAC proteins and CFP-tagged SIZ1 substrates in *N. benthamiana*. Upper panels show the subcellular localizations of fusion proteins (nuclei were imaged) and lower panels show no cell death symptoms. Scale bars, 5 μm. Similar results were obtained in three independent experiments.
(**b**) Cell death triggered by transient expression of indicated constructs. Red light emission as a result of chlorophyll fluorescence during cell death (left panel) and its quantitative measurement (right panel) are shown. Images were taken at 4 days after infiltration. Data are presented as mean ± SD (n = 17, 5, 4, 6, 3, 4, 4, 4, 8, 6, 6, 12, 4, 5, and 4 infiltrated leaves per construct from left to the right, respectively). Statistical analysis was performed using one-way ANOVA followed by Dunn's multiple comparison tests ($p < 0.05$). Similar results were obtained in three independent experiments.
(**c**) Transient expression of GFP or CFP-tagged MAC5A in combination with CFP or GFP-tagged MAC3B$^{U\text{-box-CC}}$ in *N. benthamiana*. Scale bars, 5 μm. Similar results were obtained in three independent experiments.
(**d**) Predicted intrinsic disordered region (IDR) in MAC5A predicted by D^{2}P^2, along with its protein structure predicted by AlphaFold.
(**e**) A representative transgenic *pSIZ1::SIZ1g-GFP* plant with high expression and seedling lethality phenotype. Newly germinated seedling was imaged. Similar results were obtained in ten independent transgenic lines.
(**f**) Transient expression of GFP-tagged MAC3B$^{U\text{-box-CC}}$ in combination with mCherry and SNC1-mCherry in Arabidopsis protoplasts. Scale bars, 10 μm. Similar results were obtained in three independent experiments.
(**g**) Transient expression of mCherry-tagged SNC1 in combination with free GFP, MAC5A-GFP and MAC3B$^{U\text{-box-CC}}$-GFP in *N. benthamiana*. Nuclei were imaged. Scale bars, 5 μm. Similar results were obtained in three independent experiments.

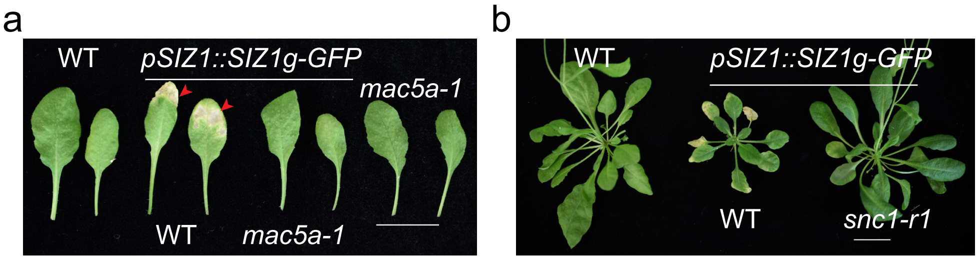

Supplementary Figure 4. MAC and SNC1 are essential for SIZ1-triggered immune activation
(a) Rosette leaves from seven-week-old plants of indicated genetic backgrounds. Scale bars, 2 cm. Similar results were obtained in three independent experiments.
(b) Seven-week-old soil-grown plants of indicated genetic backgrounds. Scale bars, 2 cm. Similar results were obtained in three independent experiments.

a

Proteomics identified SUMOylation sites in MAC proteins	
CDC5: K59, K715	NTC31: K89
MOS4: K116	MAC8: K190
SKIP: K420	MAC14: K1196
	MAC16: K2017

Predicted SUMOylation sites of MAC5A			
No.	Position	Group	Score
1	K302	LTWGR **PKPD** QDGAN	0.61
2	K110	THDSI **PKSD** VNREY	0.61
3	K212	AMKLL **GKAG** EMGTL	0.5
4	K66	RDARY **KKTE** ICQTC	0.48

Predicted SUMOylation sites of MAC3B			
No.	Position	Group	Score
1	K465	YQTAS **VKAE** WNLIK	0.93
2	K119	RVIAR **LKKE** RDEAR	0.91
3	K390	SQANV **AKFD** GHTGE	0.79
4	K54	DDIVP **IKTG** EIIKP	0.77
5	K487	GKATC **VKFG** SDAQY	0.76
6	K37	HISDY **GKCP** VTGEP	0.57
7	K430	RKLRN **FKSF** LSADA	0.5

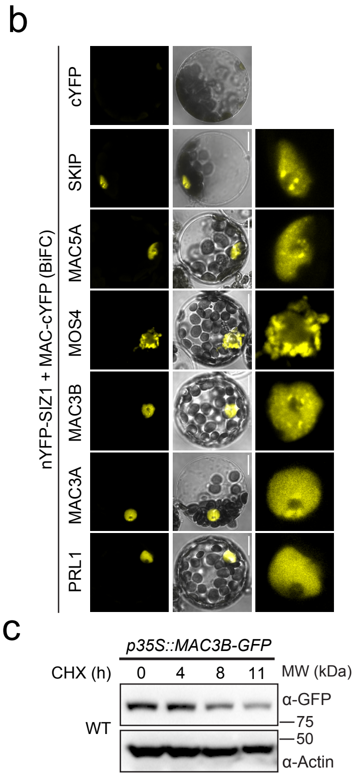

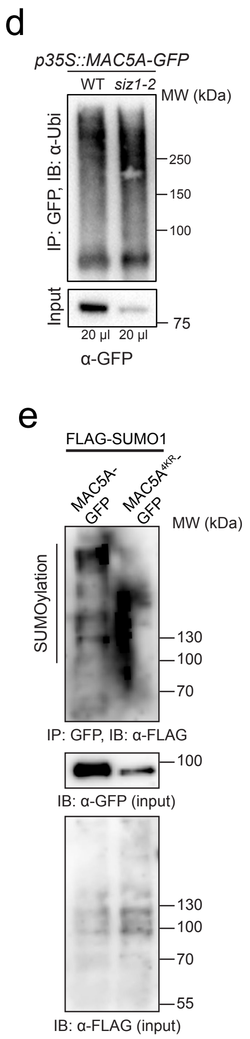

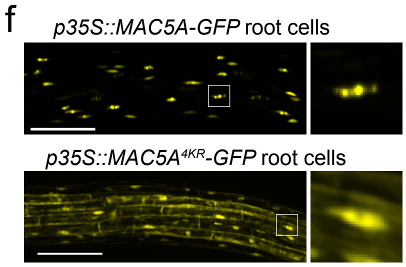

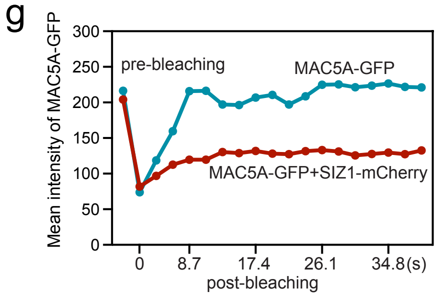

Supplementary Figure 5. SIZ1 interacts with and SUMOylates MAC proteins

(**a**) Predicted SUMOylation sites in MAC3B and MAC5A by SUMOplot™ analysis program and proteomics identified SUMOylation sites in other MAC proteins.

(**b**) Bimolecular fluorescence complementation (BiFC) assay by transiently coexpressing nYFP-SIZ1 and MAC-cYFP in Arabidopsis protoplasts. Scale bars, 10 μm. Similar results were obtained in three independent experiments.

(**c**) Cycloheximide chase experiment using *p35S::MAC3B-GFP* transgenic plants in WT background. Seedlings were treated with CHX for indicated time before sampled for immunoblotting. Similar results were obtained in three independent experiments.

(**d**) In vivo ubiquitination assay of MAC5A. Total protein from 10-day-old isogenic *p35S::MAC5A-GFP* seedlings was first immunoprecipitated with GFP-Trap and then immunoblotted with anti-ubiquitin antibody. Similar results were obtained in two independent experiments.

(**e**) In vivo SUMOylation assay of MAC5A. *p35S::FLAG-SUMO1* was transformed into protoplasts isolated from *p35S::MAC5A-GFP* or *p35S::MAC5A^{4KR}-GFP* transgenic plants. After 16 h incubation at 22°C, protoplasts were treated with 37°C for 30 min and total protein was extracted and incubated with GFP-Trap beads. IP samples were immunoblotted with anti-FLAG and anti-GFP antibodies. Vertical line indicates SUMOylated MAC5A. Similar results were obtained in two independent experiments.

(**f**) Subcellular localization of MAC5A and MAC5A^{4KR} proteins in root cells of T2 transgenic plants in WT background. Scale bars, 100 μm. Similar results were obtained in two independent transgenic lines.

(**g**) Plot showing the time course of the recovery after photobleaching of MAC5A-GFP co-expressing with or without SIZ1-mCherry in *N. benthamiana*. Time 0 indicates the start of photobleaching. Similar results were obtained in three independent experiments.
