# Supplementary material for: SUMO E3 ligase SIZ1 promotes nuclear condensate-mediated immune activation in Arabidopsis
Source: Nat Commun. 2026 Apr 15;17:5248. doi: 10.1038/s41467-026-72063-x (PMC13260931; doi:10.1038/s41467-026-72063-x)
Supplement: Supplementary file 2 — Descriptions of Additional Supplementary Files [file 41467_2026_72063_MOESM2_ESM.pdf]

Description of Additional Supplementary Files

Supplementary Data 1. Differentially expressed genes in SIZ1 overexpression lines compared to WT plants.

Supplementary Data 2. Protein candidates identified by proximity labeling proteomics using SIZ1 as bait.

Supplementary Data 3. Primers used in this study
