# Supplementary material for: SUMO E3 ligase SIZ1 promotes nuclear condensate-mediated immune activation in Arabidopsis
Source: Nat Commun. 2026 Apr 15;17:5248. doi: 10.1038/s41467-026-72063-x (PMC13260931; doi:10.1038/s41467-026-72063-x)
Supplement: Supplementary file 6 — Reporting Summary [file 41467_2026_72063_MOESM6_ESM.pdf]

nature portfolio

Corresponding author(s): Yangnan Gu

Last updated by author(s): Mar 25, 2026

Reporting Summary

Nature Portfolio wishes to improve the reproducibility of the work that we publish. This form provides structure for consistency and transparency in reporting. For further information on Nature Portfolio policies, see our Editorial Policies and the Editorial Policy Checklist.

Statistics

For all statistical analyses, confirm that the following items are present in the figure legend, table legend, main text, or Methods section.

n/a	Confirmed	
☐	☒	The exact sample size (*n*) for each experimental group/condition, given as a discrete number and unit of measurement
☐	☒	A statement on whether measurements were taken from distinct samples or whether the same sample was measured repeatedly
☐	☒	The statistical test(s) used AND whether they are one- or two-sided<br>*Only common tests should be described solely by name; describe more complex techniques in the Methods section.*
☒	☐	A description of all covariates tested
☐	☒	A description of any assumptions or corrections, such as tests of normality and adjustment for multiple comparisons
☒	☐	A full description of the statistical parameters including central tendency (e.g. means) or other basic estimates (e.g. regression coefficient) AND variation (e.g. standard deviation) or associated estimates of uncertainty (e.g. confidence intervals)
☐	☒	For null hypothesis testing, the test statistic (e.g. *F*, *t*, *r*) with confidence intervals, effect sizes, degrees of freedom and *P* value noted<br>*Give P values as exact values whenever suitable.*
☒	☐	For Bayesian analysis, information on the choice of priors and Markov chain Monte Carlo settings
☒	☐	For hierarchical and complex designs, identification of the appropriate level for tests and full reporting of outcomes
☒	☐	Estimates of effect sizes (e.g. Cohen's *d*, Pearson's *r*), indicating how they were calculated

Our web collection on statistics for biologists contains articles on many of the points above.

Software and code

Policy information about availability of computer code

Data collection	MS/MS spectra were searched against TAIR 10 database using Scaffold 5 and MSFragger 3.2 software. Reads from RNA sequencing were aligned to the reference genome of Arabidopsis (TAIR10) using Hisat2 (v2.2.051). SAMtools (v1.9.52) was used to sort and convert SAM files to BAM files.
Data analysis	GraphPad Prism 10 .4.1 was used for statistical analysis and graphics. DESeq2 (v3.14) and DEP packages (v3.20) were used for RNA analysis and proteomic analyses respectively. FIJI version 1.54j was used for measuring phenotypes and fluorescence intensities.

For manuscripts utilizing custom algorithms or software that are central to the research but not yet described in published literature, software must be made available to editors and reviewers. We strongly encourage code deposition in a community repository (e.g. GitHub). See the Nature Portfolio guidelines for submitting code & software for further information.

Data

Policy information about availability of data

All manuscripts must include a data availability statement. This statement should provide the following information, where applicable:

- Accession codes, unique identifiers, or web links for publicly available datasets
- A description of any restrictions on data availability
- For clinical datasets or third party data, please ensure that the statement adheres to our policy

Raw data files for the RNA-seq have been deposited into the NCBI GEO under accession numbers GSE307237 are publicly available. Raw data files for all mass

spectrometry analyses have been deposited to the ProteomeXchange Consortium via the PRIDE partner repository under accession number PXD067497 and are publicly available.
Disorder domain analysis was performed by D2P2 (https://d2p2.pro/).
Arabidopsis thaliana reference proteome or transcripts was obtained from TAIR (https://www.arabidopsis.org/).

Research involving human participants, their data, or biological material

Policy information about studies with [human participants or human data](). See also policy information about [sex, gender (identity/presentation), and sexual orientation]() and [race, ethnicity and racism]().

Reporting on sex and gender	not applicable
Reporting on race, ethnicity, or other socially relevant groupings	not applicable
Population characteristics	not applicable
Recruitment	not applicable
Ethics oversight	not applicable

Note that full information on the approval of the study protocol must also be provided in the manuscript.

Field-specific reporting

Please select the one below that is the best fit for your research. If you are not sure, read the appropriate sections before making your selection.

☒ Life sciences ☐ Behavioural & social sciences ☐ Ecological, evolutionary & environmental sciences

For a reference copy of the document with all sections, see nature.com/documents/nr-reporting-summary-flat.pdf

Life sciences study design

All studies must disclose on these points even when the disclosure is negative.

Sample size	No statistical methods were applied to predetermine sample sizes. Sample sizes for proximity labeling experiments (two biological replicates) and RNA-seq analyses (two biological replicates) were chosen based on standard practices in the field, as these numbers are sufficient to control for biological variability while maintaining experimental throughput. For phenotypic assays, sample sizes were determined to ensure statistical robustness: rosette size measurements ($n \geq 4$ biological samples), root length measurements ($n \geq 22$), and cell death quantification in N. benthamiana ($n \geq 6$) were selected to account for inherent physiological variation among individual plants. The exact sample sizes varied depending on the specific genotype combinations and the availability of biological material, and all sample sizes are consistent with or exceed those generally reported in similar plant molecular studies to ensure reproducibility.
Data exclusions	No data were excluded from analyses.
Replication	All data were successfully replicated and representative results were shown.
Randomization	For all experiments in this study, samples or individuals were allocated to experimental groups using randomization to minimize confounding biases and ensure unbiased representation of the underlying populations.<br>For proteomics and transcriptomics: Arabidopsis seedlings of each genotype were randomly selected from larger pools prior to sample preparation (0.5 g for proximity labeling; 0.02–0.05 g for RNA-seq) and subsequent treatments (e.g., biotin).<br>For microscopy: Cells expressing the target proteins were randomly selected for imaging and fluorescence quantification, preventing subjective selection bias.<br>For phenotypic assays: Seedlings were randomly chosen for rosette size, root length, and cell death measurements.<br>For chemical treatments (e.g., Zeocin, ABA, dexamethasone): Plants were randomly assigned to control or treatment groups to ensure comparable baseline conditions and control for confounding variables.
Blinding	Investigators were not blinded to group allocation during the experiments, as genotypes were evident during sample collection and processing. Nevertheless, the primary readouts of this study—proteomics enrichment, transcriptomic profiles, and morphometric measurements (rosette area, root length)—are quantitative parameters that were collected and analyzed in a uniform, high-throughput manner. Data analysis was performed using standardized computational pipelines and statistical tests applied consistently across all groups, which minimizes the potential for subjective bias in the final results.

Reporting for specific materials, systems and methods

We require information from authors about some types of materials, experimental systems and methods used in many studies. Here, indicate whether each material, system or method listed is relevant to your study. If you are not sure if a list item applies to your research, read the appropriate section before selecting a response.

Materials & experimental systems

n/a	Involved in the study	
☐	☒	Antibodies
☒	☐	Eukaryotic cell lines
☒	☐	Palaeontology and archaeology
☒	☐	Animals and other organisms
☒	☐	Clinical data
☒	☐	Dual use research of concern
☐	☒	Plants

Methods

n/a	Involved in the study	
☒	☐	ChIP-seq
☒	☐	Flow cytometry
☒	☐	MRI-based neuroimaging

Antibodies

Antibodies used	Anti-SUMO1 (Abcam, Ab5316); Anti-FLAG M2 (Sigma-Aldrich, F1804); Anti-Ubiquitin P4D1 (Santa Cruz, sc-8017); Anti-GFP (Clontech, Cat# 632381); Anti-Actin (Abiocode, R3772-1P); HRP-Conjugated Streptavidin (Abcam, Cat# ab7403); Goat anti-rabbit IgG-HRP secondary antibody (Thermo Scientific, Cat# 31460); Goat anti-mouse IgG-HRP secondary antibody (Thermo Scientific, Cat# 31430).
Validation	Anti-SUMO1 is polyclonal produced by recombinant full-length protein corresponding to Arabidopsis thaliana SUMO1. We used 1:500~1:1000 dilution for western blot.<br>ANTI-FLAG M2 mouse, affinity purified monoclonal antibody binds to fusion proteins containing a FLAG peptide sequence (DYKDDDDK).The antibody recognizes the FLAG peptide sequence at the N-terminus, Met-N-terminus,C-terminus, and internal sites of the fusion protein. We used 1:1000 dilution for western blot.<br>Ubiquitin (P4D1) is a mouse monoclonal antibody raised against amino acids 1-76 representing full length Ubiquitin of bovine origin. Ubiquitin (P4D1) is recommended for detection of Ubiquitin, poly-ubiquitinated and ubiquitinated proteins of mouse, rat, human and Drosophila melanogaster origin by Western Blotting (starting dilution 1:200, dilution range 1:1001:1000). We used 1:1000 dilution for western blot.<br>Anti-GFP (A monoclonal antibody produced by hybridoma cells against full-length Aequorea victoria green fluorescent protein (GFP). This antibody recognizes native and denatured forms of wild-type GFP, GFPuv, AcGFP, EGFP, destabilized EGFP variants, EBFP, EYFP, ECFP, AcGFP, and both N- and C-terminal fusion proteins containing these GFP variants in bacterial and mammalian cell lysates.) We used 1:2500 dilution for western blot. For more details: https://www.takarabio.com/products/antibodies-and-elisa/fluorescent-protein-antibodies/green-fluorescent-protein-antibodies?srsltid=AfmBOopMPnmaqCDNtZkMpcfOm6DLpN2AdRuqfrt4xtH8AZRur44e09z9.<br>Anti-Actin is Rabbit polyclonal antibodies produced by immunizing animals with a GST-fusion protein containing a fragment of the N-terminal region of Arabidopsis Actin-1 protein, which is 100% identical to the corresponding region of Oryza Actin-1 protein. This antibody was purified by the method of caprylic acid- ammonium sulfate precipitation, and is predicted to recognize the major forms of plant actin proteins.<br>HRP-Conjugated Streptavidin (Streptavidin (HRP) is a Streptomyces avidinii Full Length protein, expressed in Native, with >95% purity and suitable for Dot, IM, ICC, ELISA, IHC-P, IHC-Fr, WB.) We used 1:10000 dilution in 1% BSA for western blot. For more details: https://www.abcam.com/en-us/products/proteins-peptides/streptavidin-hrp-ab7403?srsltid=AfmBOoonIlw_qKA08Pa0r-bK7kEmhP9VcgM9W99f2kDQKU-7IkdG_qwN.

Dual use research of concern

Policy information about dual use research of concern

Hazards

Could the accidental, deliberate or reckless misuse of agents or technologies generated in the work, or the application of information presented in the manuscript, pose a threat to:

No	Yes	
☒	☐	Public health
☒	☐	National security
☒	☐	Crops and/or livestock
☒	☐	Ecosystems
☒	☐	Any other significant area

Experiments of concern

Does the work involve any of these experiments of concern:

No	Yes	
☒	☐	Demonstrate how to render a vaccine ineffective
☒	☐	Confer resistance to therapeutically useful antibiotics or antiviral agents
☒	☐	Enhance the virulence of a pathogen or render a nonpathogen virulent
☒	☐	Increase transmissibility of a pathogen
☒	☐	Alter the host range of a pathogen
☒	☐	Enable evasion of diagnostic/detection modalities
☒	☐	Enable the weaponization of a biological agent or toxin
☒	☐	Any other potentially harmful combination of experiments and agents

Plants

Seed stocks

All Arabidopsis plants used in this study are in the Columbia-0 background. The ka120 (Salk_148803), siz1-2 (Salk_065397), siz1-3 (Salk_ Salk_034008), prl1-2 (Salk_039427), mac3a mac3b (Salk_089300, Salk_050811), mos4-1 (CS69914), mac5a-1 (Salk_132881), mac5a-2 (Salk_142085), and abi5 (Salk_013163) mutants were obtained from Arabidopsis Biological Resource Center (ABRC). The snc1-r1 was obtained from Yuelin Zhang lab and Xinnian Dong lab, respectively.
Transgenic plants were produced via Agrobacterium tumefaciens (strain GV3103)-mediated floral dip transformation. Transgenic lines were selected on media containing appropriate antibiotics: Basta (25 µg/ml, Supelco), kanamycin A (50 µg/ml, Gold Biotechnology), or hygromycin B (50 µg/ml, Invitrogen).
For each transgenic construct, multiple independent transgenic lines were generated and analyzed to confirm phenotypic reproducibility and rule out positional effects. The specific generations used for each experiment were as follows:
T1 generation: Figure 1d and 1e; T2 generation: Figure 1f and Extended Data Figure 2a; T3 generation: Figure 3f, 3h, Figure 4, Extended Data Figure 1d, 1e, 1h, 4a; T4 generation: Figure 5

Novel plant genotypes

All the higher order mutants in this study were obtained through genetic crosses.

Authentication

All mutant lines used in this study were generated in the Col-0 ecotype background and were backcrossed with wild-type Col-0 to remove unlinked mutations. Genotypes were confirmed by PCR (primers in Supplementary Table 3).
To ensure the reliability of phenotypic observations: Multiple independent lines and, where applicable, different tagging strategies were analyzed to confirm that phenotypes were consistent and not due to positional effects or second-site mutations. The expression of transgenes was verified by detecting fluorescence or by western blot analysis to confirm protein production.
For T-DNA insertion lines, we assessed segregation patterns to identify lines with single insertions, thereby minimizing the risk of confounding effects from second-site insertions. Mosaicism was not observed in the generations used for experiments, as stable homozygous lines were confirmed by genotyping.
