# Supplementary material for: SUMO E3 ligase SIZ1 promotes nuclear condensate-mediated immune activation in Arabidopsis
Source: Nat Commun. 2026 Apr 15;17:5248. doi: 10.1038/s41467-026-72063-x (PMC13260931; doi:10.1038/s41467-026-72063-x)
Supplement: Supplementary file 7 — Transparent Peer Review file [file 41467_2026_72063_MOESM7_ESM.pdf]

nature portfolio

Peer Review File

SUMO E3 ligase SIZ1 promotes nuclear condensate-mediated immune activation in Arabidopsis

Corresponding Author: Professor Yangnan Gu

This file contains all reviewer reports in order by version, followed by all author rebuttals in order by version.

Version 0:

Reviewer comments:

Reviewer #1

(Remarks to the Author)

This manuscript presents an interesting discovery regarding the positive immune functions of SIZ1 and the role of SUMOylation in condensate-mediated immune responses. The authors describe a surprising immune activation function for SIZ1. The manuscript is generally well written, with good logical flow. In addition, the manuscript references transcriptomic and proxiome data that will likely be highly useful to the research community. However, I have some concerns regarding data interpretation and biological relevance, as described in the following points.

Writing and data presentation:
1. The title is over-claiming; “Arabidopsis” should be used instead of “plants”.
2. To be consistent with current literature, TNL should be used instead of TIR-NLR.
3. In some data figures, the authors label “siz1”. As both siz1-2 and siz1-3 are used at times, the full allele name should be written at all times to avoid confusion.

Data analysis and interpretation:
4. Why was a Chi-square goodness-of-fit test used for the statistical analysis in Figure 1c? Is a one-sample t-test not more appropriate? There is also a rendering issue with the cpr5-1 bar in this figure.
5. I do not follow the authors’ logic in concluding “SIZ1’s pro-immune function is already activated in the ka120 background” (Line 131). In this scenario, autoimmunity of ka120 would be at least partially attributed to enhanced SIZ1 activity. However, Category 0 and II SIZ1 overexpression line display autoimmunity that appears to be considerably stronger than ka120. Consequently, overexpressing SIZ1 to similar levels in ka120 should result in similar, if not stronger, autoimmunity. Instead, the data presented here suggests that KA120 is required for SIZ1 immune induction. This data needs reconciling with the author’s model, in which SIZ1 and KA120 play opposing functions in MDNC-mediated immunity.

Biological relevance:
6. As noted in the discussion (Line 324), the authors do not provide evidence of increased MAC protein SUMOylation upon pathogen infection, citing “the relatively small proportion of infected cells” as a limiting factor. This may be overcome by using transgenic expression of AvrRps4, allowing for immune activation in a higher proportion of cells. Demonstrating increased MAC SUMOylation under these conditions would offer the biological relevance of this study.
7. The biological relevance of this study is also hampered by the reliance on MAC overexpression in At or transient expression in Nb to induce SIZ1 and SNC1 recruitment to nuclear condensates (Figure 3h, i). Can this phenomenon be observed without MAC overexpression? The model proposes that the autoimmunity of SIZ1 overexpression lines is caused by SIZ1 recruitment to MDNCs. In this case, autoimmune SIZ1-GFP lines in WT background should display localisation of SIZ1 to nuclear puncta. The authors should also include fluorescence images from the pSIZ1g::SIZ1-GFP transgenic lines.
8. The biological significance of the sumo sites identified in the MAC complex components should be tested by examining the respective point mutants. Are those sites conserved, and can the point mutants complement the mac mutants, are the mutants showing altered stability or function, and etc.?
9. Based on the authors’ model, SIZ1 is a sumo E3, and the MAC complex is involved in splicing, the immune activating activities in the condensates comes from TNL SNC1. Thus loss-of-function snc1 mutant should be used to test whether the SIZ1 overexpression mediated autoimmunity relies on SNC1. The proxiome of SIZ1 should also be examined in loss-of-function snc1 mutant background to validate the model.

10. The authors have not excluded the possibility that SNC1 is merely guarding SIZ1 overexpression, or guarding the MAC complex during immune activation.

Reviewer #2

(Remarks to the Author)
Previously, it has been shown that loss-of-function mutations in SUMO E3 ligase SIZ1 result in autoimmunity. In this manuscript, the authors found that overexpression of SIZ1 activates immune response. Based on several lines of evidence, the authors proposed a model in which pathogen infection induces SIZ1 upregulation, leading to enhanced SUMOylation of MAC proteins and increased protein stability, which in turn increases their condensation propensity and maintains their high local concentrations within MDNCs. This, in turn, stabilizes the condensate scaffold and supports MDNC-mediated immune signaling. Overall, the study provides very interesting findings and will be of great interest to plant biology field. The experiments were well done, and data were reasonably analyzed, which support their arguments. However, some issues should be addressed to improve the manuscript.

Comments:
1. My major concern is whether the immune response induced by SIZ1 overexpression really exists in nature. As the authors mentioned in the discussion part, it is necessary to show that pathogen infection promotes SUMOylation of MAC proteins in WT but not in the siz1 loss-of-function mutant.
2. The authors should provide more evidence to show that SUMOylation of MAC proteins increases their protein stability, condensation propensity, and high local concentrations within MDNCs. To this end, it is necessary to characterize the SUMOylation site(s) of MAC proteins and determine whether K-to-R mutation affects protein stability, ubiquitination, and condensation of MAC proteins within MDNCs.
3. In Fig. 5C, the +MG132 siz1-2 control is missing. Moreover, to support that SUMOylation of MAC promotes its protein stability, it is necessary to perform a protein decay assay (using Cyclohexamide, with or without MG132).

Reviewer #3

(Remarks to the Author)
Comments on Jia et al.

This is an elegant and well-executed study that connects protein condensates to autoimmunity in plants. The authors show that overexpression of the SUMO E3 ligase SIZ1 activates immune responses and cell death, an intriguing contrast to the well-documented siz1 loss-of-function phenotype. Indeed, complex genetic background often complicates functional interpretation. This is a good example. I congratulate the authors for a clean dissection of both mutant phenotypes. Through powerful genetic and biochemical analyses, they demonstrate that SIZ1 interacts with and SUMOylates MAC complex components, stabilizing them in vivo. Moreover, SIZ1 and immune receptors such as SNC1 localize to MAC condensates, thereby potentiating plant defense. The manuscript is clearly written, and the findings will be of strong interest to both the condensate biology and plant immunity communities. I have a few comments aimed at strengthening the mechanistic interpretation and overall conclusion:

It remains unclear how SIZ1 mechanistically promotes MAC condensate–mediated immune activation. The authors show that MAC components are SIZ1 substrates and that their SUMOylation levels are reduced in the siz1 loss-of-function mutant. However, most phenotypes described here arise from SIZ1 overexpression, not loss of function. It is therefore possible that SIZ1 substrates are already SUMOylated to saturation under normal conditions. Assessing the SUMOylation status and stability of MAC components in the SIZ1-OX background would clarify whether overexpressed SIZ1 further enhances substrate modification or acts through an alternative mechanism.

Does SIZ1 overexpression alter MAC condensation behavior? The authors' transient expression system provides a convenient platform to test this. Quantitative analyses of condensate number, size, and material properties in the presence or absence of SIZ1 overexpression would directly address whether SIZ1 modulates the physical assembly or stability of MAC condensates. Such experiments would significantly strengthen the central conclusion that SIZ1 regulates immunity through condensate remodeling.

Reviewer #4

(Remarks to the Author)
In this study, Jia et al. reported findings that differ from previous understandings, showing that SIZ1 cannot be simply defined as a negative regulator of plant immunity. Instead, SIZ1 can also act as a positive regulator that potentiates immune responses.
Through extensive genetic and cell biological evidence, the authors demonstrate that the SUMO E3 ligase SIZ1 promotes nuclear condensate–mediated immune activation in plants, highlighting the complexity of SIZ1 functions and suggesting that its diverse roles may depend on distinct substrates. While this work challenges the traditional viewpoint and provides convincing evidence and reasoning that broaden our understanding of SIZ1's roles, several issues should be addressed to strengthen the manuscript.

1. All experiments in this study used pSIZ1::SIZ1g–GFP to induce cell death. Have the authors tested whether the untagged construct (pSIZ1::SIZ1g) can also trigger cell death? This control would clarify whether the observed phenotype is due to the GFP tag.
2. In Figures 1d and 1e, the SIZ1–GFP transcript levels vary substantially among different pSIZ1::SIZ1g–GFP transgenic lines, correlating with the severity of the necrotic phenotype. A key question is whether this phenotype results from insertional effects, such as disruption of endogenous genes, rather than SIZ1 overexpression per se. Moreover, as RNA data only partially reflect expression differences, the authors are encouraged to present protein-level evidence (e.g., immunoblot of SIZ1–GFP) to more directly demonstrate expression variation among lines.
3. In Figure 1g, SIZ1g induces stronger necrosis than SIZ1c. How do the authors explain this? Is the protein accumulation level of SIZ1g higher than that of SIZ1c?
4. Line 274: Does the spontaneous necrosis observed in the siz1 mutant affect the accumulation of MAC proteins?
5. In Figure 3f, after pathogen treatment, MAC3B-mediated condensates were observed in guard cells. Do similar condensates occur in non-guard cells? Does SIZ1 undergo phase separation in non-guard cells as well?
6. In Figure 3f, pathogen-induced phase separation of SIZ1 was observed in plants co-expressing MAC3B. Can pathogen infection alone trigger SIZ1 condensate formation in plants overexpressing SIZ1 only (without MAC3B co-expression)?
7. In Figure 3h, the condensate pattern (single fluorescent dot) appears different from that shown in Figure 3e (multiple fluorescent dots). Please clarify or discuss this discrepancy.
8. In Figure 4c, a p35S::KA120–YFP transgenic control is missing and should be included for comparison.
9. In Figure 5b and Extended Data Figure 5a, do the ubiquitination levels of MAC3B and MOS4 change in the siz1 mutant background? A key mechanistic question is how SIZ1-mediated SUMOylation interferes with MAC5A ubiquitination to enhance its stability. Additional genetic and biochemical evidence—such as constructing MAC5A point mutants defective in SUMOylation—would strengthen the conclusion that SUMO modification affects MAC5A ubiquitination and stability.
10. In Extended Data Figure 1d, were there any pSIZ1g::SIZ1–GFP/siz1 complementation lines that displayed necrosis? If so, is this phenotype correlated with the expression level of SIZ1–GFP?
11. In Extended Data Figure 1j, SIZ1 mRNA levels increased under all tested treatments. Was a corresponding increase observed at the protein level?
12. In both SIZ1 overexpression and knockout backgrounds, how do MAC-dependent nuclear condensates (MDNCs) differ in number or persistence?
13. Do the nuclear condensates shown in this study exhibit fluorescence recovery after photobleaching (FRAP), indicating liquid-like behavior?
14. The reviewer is also interested in whether the necrosis-inducing function of SIZ1 is conserved across species. Given the high evolutionary conservation of SIZ1, it would be worthwhile to examine whether overexpression of NbSIZ1 in Nicotiana benthamiana also triggers cell death, as observed in Arabidopsis.

Version 1:

Reviewer comments:

Reviewer #1

(Remarks to the Author)
The authors have done a thorough job addressing almost all my concerns. This is now a nice story ready to be published. Congratulations to the authors on a nice piece of work!

Reviewer #2

(Remarks to the Author)
My concerns have been adequately addressed in this version and it is now a much better story.

Reviewer #3

(Remarks to the Author)
The authors have addressed all my concerns and I have no further questions.

Reviewer #4

(Remarks to the Author)
The author has addressed my concerns in a satisfied manner. I suggest accept.

Open Access This Peer Review File is licensed under a Creative Commons Attribution 4.0 International License, which permits use, sharing, adaptation, distribution and reproduction in any medium or format, as long as you give appropriate credit to the original author(s) and the source, provide a link to the Creative Commons license, and indicate if changes were made.
In cases where reviewers are anonymous, credit should be given to 'Anonymous Referee' and the source.
The images or other third party material in this Peer Review File are included in the article's Creative Commons license, unless indicated otherwise in a credit line to the material. If material is not included in the article's Creative Commons license and your intended use is not permitted by statutory regulation or exceeds the permitted use, you will need to obtain permission directly from the copyright holder.
To view a copy of this license, visit https://creativecommons.org/licenses/by/4.0/

Remarks to the Authors / Authors' Responses

Reviewer #1

This manuscript presents an interesting discovery regarding the positive immune functions of SIZ1 and the role of SUMOylation in condensate-mediated immune responses. The authors describe a surprising immune activation function for SIZ1. The manuscript is generally well written, with good logical flow. In addition, the manuscript references transcriptomic and proxiome data that will likely be highly useful to the research community. However, I have some concerns regarding data interpretation and biological relevance, as described in the following points.

We thank the Reviewer's careful evaluation of the work and the constructive feedback. Below, we address the concerns raised.

Writing and data presentation:

1. The title is over-claiming; "Arabidopsis" should be used instead of "plants".

We replaced "plants" with "Arabidopsis" in the title.

2. To be consistent with current literature, TNL should be used instead of TIR-NLR.

TIR-NLR is replaced by TNL across the main text.

3. In some data figures, the authors label "siz1". As both siz1-2 and siz1-3 are used at times, the full allele name should be written at all times to avoid confusion.

The full allele names of all *siz1* mutants used are specified in the revised manuscript now.

Data analysis and interpretation:

4. Why was a Chi-square goodness-of-fit test used for the statistical analysis in Figure 1c? Is a one-sample t-test not more appropriate? There is also a rendering issue with the cpr5-1 bar in this figure.

Because the data type is continuous rather than categorical, we agree with the Reviewer that t-test is more appropriate in this case than the chi-square goodness-of-fit test. The statistics has been redone and the graphing issue was also solved.

5. I do not follow the authors' logic in concluding "SIZ1's pro-immune function is already activated in the ka120 background" (Line 131). In this scenario, autoimmunity of ka120 would be at least partially attributed to enhanced SIZ1 activity. However, Category 0 and II SIZ1 overexpression line display autoimmunity that appears to be considerably stronger than ka120. Consequently, overexpressing SIZ1 to similar levels in ka120 should result in similar, if not stronger, autoimmunity. Instead, the data presented here suggests that KA120 is required for SIZ1 immune induction. This data needs reconciling with the author's model, in which SIZ1 and KA120 play opposing functions in MDNC-mediated immunity.

Nice observation! Yes, we have previously considered this interpretation (KA120 is required for SIZ1 immune induction), which is totally logical based on the SIZ1 OE/*ka120* phenotype. However, subsequent analysis revealed that KA120 overexpression could rescue the SIZ1-OE–induced immune activation (Fig. 4b), providing more compelling evidence that KA120 suppresses SIZ1-mediated immune activation. Therefore, an alternative explanation for the SIZ1 OE/*ka120* phenotype is that SIZ1-induced immunity engages the same signaling pathway that becomes active upon

KA120 loss. Note that loss of *KA120* appears to cap the overall severity of the SIZ1-OE phenotype, suggesting that KA120 may also modulate SIZ1 activity, such as through regulating its expression level or post-translational activity, a layer of control that we are working on but will not be incorporated into the model in this paper.

Biological relevance:

6. As noted in the discussion (Line 324), the authors do not provide evidence of increased MAC protein SUMOylation upon pathogen infection, citing “the relatively small proportion of infected cells” as a limiting factor. This may be overcome by using transgenic expression of AvrRps4, allowing for immune activation in a higher proportion of cells. Demonstrating increased MAC SUMOylation under these conditions would offer the biological relevance of this study.

Since receiving the reviewers’ feedback, we have worked to optimize the experimental conditions to detect MAC3B SUMOylation upon pathogen infection. We increased both the pathogen inoculum concentration using infiltration and seedling incubation inoculation; however, consistent with our previous results, detection of MAC3B SUMOylation remained challenging.

We made significant progress when we increased the inoculation temperature to 28°C, which elevated basal protein SUMOylation levels and enabled detection of MAC3B SUMOylation by IP followed by anti-SUMO immunoblotting. Under this condition, MAC3B SUMOylation was significantly enhanced upon *Pst*DC3000/AvrRPS4 infection, and this modification was dependent on SIZ1 (see results shown on the right). Even though 28°C may partially compromise TNL immune output, the results strongly support our hypothesis. These data are now included in the new Figure 5d.

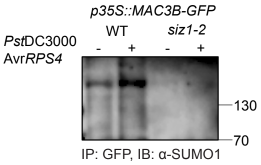

7. The biological relevance of this study is also hampered by the reliance on MAC overexpression in At or transient expression in Nb to induce SIZ1 and SNC1 recruitment to nuclear condensates (Figure 3h, i). Can this phenomenon be observed without MAC overexpression? The model proposes that the autoimmunity of SIZ1 overexpression lines is caused by SIZ1 recruitment to MDNCs. In this case, autoimmune SIZ1-GFP lines in WT background should display localization of SIZ1 to nuclear puncta. The authors should also include fluorescence images from the pSIZ1g::SIZ1-GFP transgenic lines.

Yes, SIZ1 does form strong nuclear condensates in *pSIZ1::SIZ1g-GFP* transgenic lines even without MAC overexpression (WT background, see representative images on the right). Importantly, all *pSIZ1::SIZ1g-GFP* lethal lines display these condensates. This data is now included in the new Extended Data Figure 3e.

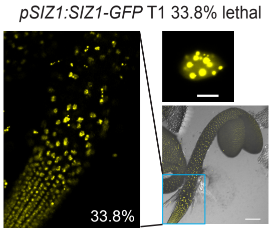

8. The biological significance of the sumo sites identified in the MAC complex components should be tested by examining the respective point mutants. Are those sites conserved, and can the point mutants complement the mac mutants, are the mutants showing altered stability or function, and etc.?

We predicted potential SUMOylation sites in two major MAC proteins investigated in this study, MAC5A and MAC3B, using SUMO-GPS and SUMOplot™. Four lysine residues with the highest prediction scores were mutated to arginine in each protein. For MAC5A, this quadruple mutation MAC5A^{4KR} almost completely abolished SUMOylation compared to the wild-type protein (see figure below, panel a). The MAC5A^{4KR} mutant exhibited an obvious change in condensation capacity: while the wild-type protein localized exclusively to the nucleus and nuclear condensates, the mutant failed to form condensates (panel b). The data were added to the new Extended Data Figure 5e and 5f.

Interestingly, MAC5A^{4KR} exhibited increased stability compared with the WT protein (panel c). Because lysine residues can be targets of both SUMOylation and ubiquitination, and these modifications can have opposing effects on MAC5A stability in this case, therefore, using protein stability as a readout of SUMOylation effect is potentially confounded in this context. This duality complicates interpretation of results obtained using MAC^{KR} mutants and highlights the potential interplay between SUMOylation- and ubiquitination-dependent regulation of MAC activity, which warrants future investigation. This complexity was added to the Discussion session.

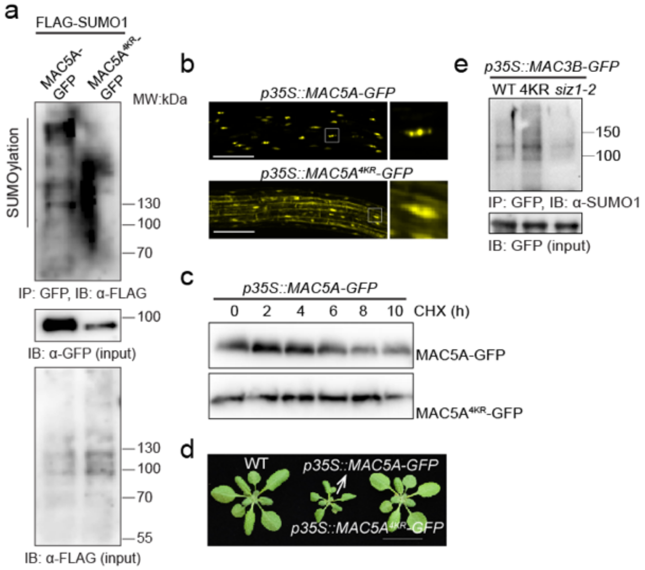

Furthermore, while overexpression of MAC5A trigged an autoimmunity phenotype, the MAC5A^{4KR} mutant did not (panel d). These results support our hypothesis that SUMOylation promotes the formation of MAC-associated nuclear condensates and is required for their function.

We also tried MAC3B but found that the MAC3B^{4KR} mutant retained SUMOylation (panel e), suggesting that additional SUMO-acceptor lysine residues in MAC3B, distinct from those predicted, are responsible for SIZ1-mediated SUMOylation.

9. Based on the authors' model, SIZ1 is a sumo E3, and the MAC complex is involved in splicing, the immune activating activities in the condensates comes from TNL SNC1. Thus loss-of-function snc1 mutant should be used to test whether the SIZ1 overexpression mediated autoimmunity relies on SNC1. The proxiome of SIZ1 should also be examined in loss-of-function snc1 mutant background to validate the model.

We thank the reviewer for this suggestion, which addresses a key point we had begun investigating prior to the initial submission. We crossed the cell death-inducing *pSIZ1::SIZ1g-GFP* line into the *snc1-r1* loss-of-function mutant background. Our results show that the *snc1-r1* mutation completely suppresses the constitutive cell death and autoimmune phenotypes caused by SIZ1 overexpression. This genetic data supports our model that SIZ1-mediated autoimmunity depends on the immune receptor SNC1 and was added to new Extended Data Figure 4b.

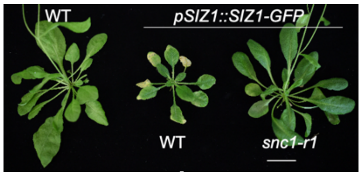

Since SNC1 is considered a client, rather than a scaffold, in MAC condensates, we do not expect the SIZ1 proxiome to differ substantially between *snc1* loss-of-function background and WT.

10. The authors have not excluded the possibility that SNC1 is merely guarding SIZ1 overexpression, or guarding the MAC complex during immune activation.

We appreciate the reviewer's question regarding a potential guarding relationship. SNC1 has been reported to guard ADR1 through direct protein–protein interactions (Wang et al., 2023). However, we were unable to detect a physical interaction between SNC1 and SIZ1 by coIP, suggesting that SNC1 may not directly guard SIZ1. The possibility that SNC1 guards MDNC is an intriguing hypothesis, especially given the rescue of SIZ1-induced cell death by *snc1-r1*, and worthy of future investigation. However, this model is not mutually exclusive with our current findings, as the SIZ1–MAC condensation described here could operate upstream of this guarding mechanism.

Reviewer #2

Previously, it has been shown that loss-of-function mutations in SUMO E3 ligase SIZ1 result in autoimmunity. In this manuscript, the authors found that overexpression of SIZ1 activates immune response. Based on several lines of evidence, the authors proposed a model in which pathogen infection induces SIZ1 upregulation, leading to enhanced SUMOylation of MAC proteins and increased protein stability, which in turn increases their condensation propensity and maintains their high local concentrations within MDNCs. This, in turn, stabilizes the condensate scaffold and supports MDNC-mediated immune signaling. Overall, the study provides very interesting findings and will be of great interest to plant biology field. The experiments were well done, and data were reasonably analyzed, which support their arguments. However, some issues should be addressed to improve the manuscript.

We thank this reviewer's positive comments and addressed your concerns below.

Comments:

1. My major concern is whether the immune response induced by SIZ1 overexpression really exists in nature. As the authors mentioned in the discussion part, it is necessary to show that pathogen infection promotes SUMOylation of MAC proteins in WT but not in the siz1 loss-of-function mutant.

Since receiving the reviewers' feedback, we have worked to optimize the experimental conditions to detect MAC3B SUMOylation upon pathogen infection. We increased both the pathogen inoculum concentration using infiltration and seedling incubation inoculation; however, consistent with our previous results, detection of MAC3B SUMOylation remained challenging.

We made significant progress when we increased the inoculation temperature to 28°C, which elevated basal protein SUMOylation levels and enabled detection of MAC3B SUMOylation by IP followed by anti-SUMO immunoblotting. Under this condition, MAC3B SUMOylation was significantly enhanced upon *Pst*DC3000/AvrRPS4 infection, and this modification was dependent on SIZ1 (see results shown on the right). Even though 28°C may partially compromise TNL immune output, the results strongly support our hypothesis. These data are now included in the new Figure 5d.

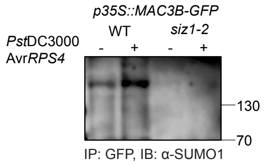

2. The authors should provide more evidence to show that SUMOylation of MAC proteins increases their protein stability, condensation propensity, and high local concentrations within MDNCs. To this end, it is necessary to characterize the SUMOylation site(s) of MAC proteins and determine whether K-to-R mutation affects protein stability, ubiquitination, and condensation of MAC proteins within MDNCs.

Following the Reviewer's suggestion, we characterized SUMO mutants of MAC proteins. We predicted potential SUMOylation sites in two major MAC proteins investigated in this study, MAC5A and MAC3B, using SUMO-GPS and SUMOplot™. Four lysine residues with the highest prediction scores were mutated to arginine in each protein. For MAC5A, this quadruple mutation MAC5A^{4KR} almost completely abolished SUMOylation compared to the wild-type protein (see figure below, panel a). The MAC5A^{4KR} mutant exhibited an obvious change in condensation capacity: while the wild-type protein localized exclusively to the nucleus and nuclear condensates, the mutant failed to form condensates (panel b). The data were added to the new Extended Data Figure 5e and 5f.

Interestingly, MAC5A^{4KR} exhibited increased stability compared with the WT protein (panel c). Because lysine residues can be targets of both SUMOylation and ubiquitination, and these modifications can have opposing effects on MAC5A stability in this case, therefore, using protein stability as a readout of SUMOylation effect is potentially confounded in this context. This duality complicates interpretation of results obtained using MAC^{KR} mutants and highlights the potential

interplay between SUMOylation- and ubiquitination-dependent regulation of MAC activity, which warrants future investigation. This complexity was added to the Discussion session.

Furthermore, while overexpression of MAC5A trigged an autoimmunity phenotype, the MAC5A^{4KR} mutant did not (panel d). These results support our hypothesis that SUMOylation promotes the formation of MAC-associated nuclear condensates and is required for their function.

We also tried MAC3B but found that the MAC3B^{4KR} mutant retained SUMOylation (panel e), suggesting that additional SUMO-acceptor lysine residues in MAC3B, distinct from those predicted, are responsible for SIZ1-mediated SUMOylation.

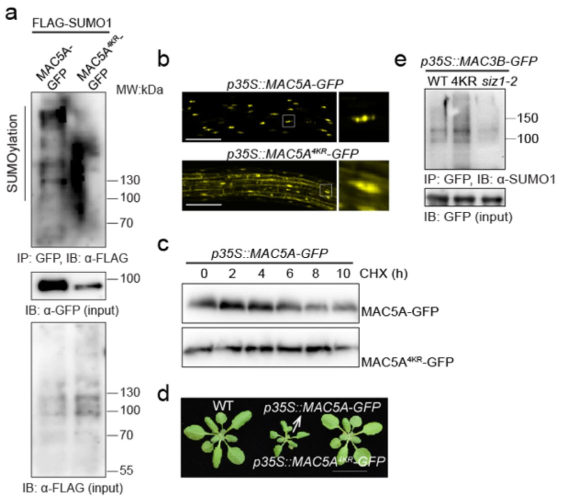

3. In Fig. 5C, the +MG132 siz1-2 control is missing. Moreover, to support that SUMOylation of MAC promotes its protein stability, it is necessary to perform a protein decay assay (using Cyclohexamide, with or without MG132).

Thanks for the suggestion. The +MG132 siz1-2 control for MAC3B and MAC5A is now added to replace the previous data in Fig. 5c (shown in the left panel below).

To demonstrate that the altered protein accumulation reflects changes in protein degradation, we performed the suggested CHX chase assay using MAC3B and MOS4 in a time-course experiment. The results clearly show that, in the absence of de novo protein synthesis, MAC proteins undergo constitutive degradation (right panel below). For space issue, we included only the MAC3B data in the new Extended Data Figure 5c.

However, interpretation became more complicated when MG132 and CHX were applied together, as we observed variability at later time points. We suspect this is due to the cytotoxic effects of prolonged dual treatment, which begin to cause noticeable cell death in seedlings after ~8 hours. Since MG132 treatment alone produces a clear, SIZ1-dependent effect, whereas the combined MG132+CHX treatment is confounded by cytotoxicity, we believe the current data sufficiently address the reviewer's concern.

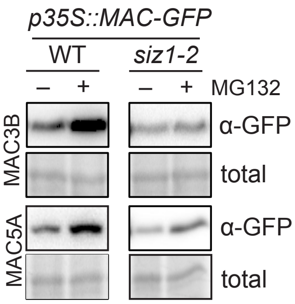

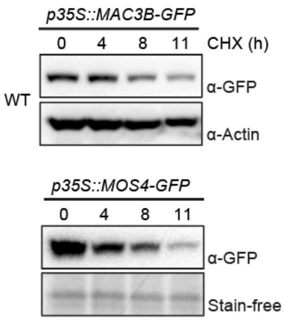

Reviewer #3 (Remarks to the Author)

This is an elegant and well-executed study that connects protein condensates to autoimmunity in plants. The authors show that overexpression of the SUMO E3 ligase SIZ1 activates immune responses and cell death, an intriguing contrast to the well-documented siz1 loss-of-function phenotype. Indeed, complex genetic background often complicates functional interpretation. This is a good example. I congratulate the authors for a clean dissection of both mutant phenotypes. Through powerful genetic and biochemical analyses, they demonstrate that SIZ1 interacts with and SUMOylates MAC complex components, stabilizing them in vivo. Moreover, SIZ1 and immune receptors such as SNC1 localize to MAC condensates, thereby potentiating plant defense. The manuscript is clearly written, and the findings will be of strong interest to both the condensate biology and plant immunity communities. I have a few comments aimed at strengthening the mechanistic interpretation and overall conclusion:

We appreciate this reviewer's generous comments and recognition of the work. Please see our responses to specific comments below.

It remains unclear how SIZ1 mechanistically promotes MAC condensate–mediated immune activation. The authors show that MAC components are SIZ1 substrates and that their SUMOylation levels are reduced in the siz1 loss-of-function mutant. However, most phenotypes described here arise from SIZ1 overexpression, not loss of function. It is therefore possible that SIZ1 substrates are already SUMOylated to saturation under normal conditions. Assessing the SUMOylation status and stability of MAC components in the SIZ1-OX background would clarify whether overexpressed SIZ1 further enhances substrate modification or acts through an alternative mechanism.

We thank the reviewer for this excellent question. Rather than using a SIZ1-OX background to assess MAC SUMOylation status, we took a more direct and biological relevant approach. We optimized pathogen inoculation conditions at a slightly elevated temperature and examined whether MAC proteins exhibit enhanced SUMOylation upon immune induction. Our results show that AvrRPS4-triggered immune activation significantly increases MAC3B SUMOylation (see data on the right), indicating that MAC3B SUMOylation is not saturated prior to immune induction. Importantly, this enhancement is SIZ1-dependent, supporting the requirement of SIZ1 for immune-induced MAC3B SUMOylation and consistent with the model we propose. This data was added to the new Figure 5d.

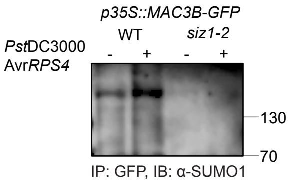

Does SIZ1 overexpression alter MAC condensation behavior? The authors' transient expression system provides a convenient platform to test this. Quantitative analyses of condensate number, size, and material properties in the presence or absence of SIZ1 overexpression would directly address whether SIZ1 modulates the physical assembly or stability of MAC condensates. Such experiments would significantly strengthen the central conclusion that SIZ1 regulates immunity through condensate remodeling.

Thanks for your suggestions. We quantified the MAC5A condensates number with or without overexpressing SIZ1-mcherry in tobacco transient expression system. When MAC5A is expressed alone, nearly 40% of cells exhibited only one or two enlarged condensates. In contrast, co-expression with SIZ1-mCherry led to a significant increase in condensate number per cell (see results shown on the right, panel a).

We further predicted potential SUMOylation sites in MAC5A using SUMO-GPS and SUMOplot™. Four lysine residues with the highest

a

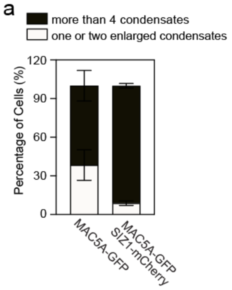

prediction scores were mutated to arginine in each protein. This quadruple mutant (MAC5A^{4KR}) almost completely abolished SUMOylation compared to the wild-type protein (panel b). The MAC5A^{4KR} mutant exhibited an obvious change in condensation capacity: while the wild-type protein localized exclusively to the nucleus and nuclear condensates, the mutant failed to form condensates (panel c). This result supports our hypothesis that SUMOylation promotes the formation of MAC-associated nuclear condensates. The data were added to the new Extended Data Figure 5e and 5f.

To determine if SIZ1 also alters the material properties of these condensates, we performed FRAP. We found that the MAC5A fluorescence recovery was significantly reduced upon coexpression with SIZ1 (panel d), indicating that SUMOylated MAC is more stably incorporated or interlocked within the condensates, consistent with the proposed model. The data was added to the new Extended Data Figure 5g.

Together, these results demonstrate that SIZ1 influences both the morphology (size and number) and the dynamic properties of MAC5A condensates.

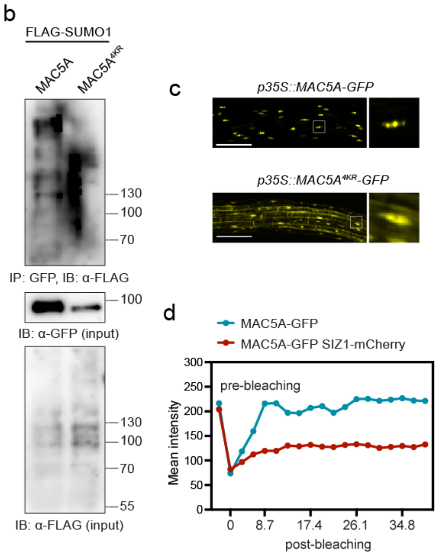

Reviewer #4 (Remarks to the Author):

In this study, Jia et al. reported findings that differ from previous understandings, showing that SIZ1 cannot be simply defined as a negative regulator of plant immunity. Instead, SIZ1 can also act as a positive regulator that potentiates immune responses.
Through extensive genetic and cell biological evidence, the authors demonstrate that the SUMO E3 ligase SIZ1 promotes nuclear condensate–mediated immune activation in plants, highlighting the complexity of SIZ1 functions and suggesting that its diverse roles may depend on distinct substrates. While this work challenges the traditional viewpoint and provides convincing evidence and reasoning that broaden our understanding of SIZ1's roles, several issues should be addressed to strengthen the manuscript.

1. All experiments in this study used pSIZ1::SIZ1g–GFP to induce cell death. Have the authors tested whether the untagged construct (pSIZ1::SIZ1g) can also trigger cell death? This control would clarify whether the observed phenotype is due to the GFP tag.

We have showed that *pSIZ1::SIZ1g-TurboID* lines exhibit similar cell death phenotypes similar to the GFP lines in Arabidopsis (Fig. S2), indicating the effect is not caused by the GFP tag. To further address the reviewer's concern, we also constructed an untagged *pSIZ1::SIZ1g* construct and confirmed that it is capable of inducing strong cell death in *N. benthamiana* (shown on the right).

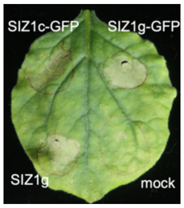

2. In Figures 1d and 1e, the SIZ1–GFP transcript levels vary substantially among different pSIZ1::SIZ1g–GFP transgenic lines, correlating with the severity of the necrotic phenotype. A key question is whether this phenotype results from insertional effects, such as disruption of endogenous genes, rather than SIZ1 overexpression per se. Moreover, as RNA data only partially reflect expression differences, the authors are encouraged to present protein-level evidence (e.g., immunoblot of SIZ1–GFP) to more directly demonstrate expression variation among lines.

Regarding the first point, we acknowledge that using a single line could raise concerns about insertional effects. However, the observed correlation between phenotype severity and expression levels was established across multiple independent lines, making it highly unlikely that the phenotype results from insertional artifacts.

To address the second point, we quantified SIZ1-GFP fluorescence in these independent lines, which show consistent protein expression trend with their transcript levels and phenotype severity (shown on the right).

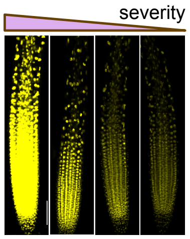

3. In Figure 1g, SIZ1g induces stronger necrosis than SIZ1c. How do the authors explain this? Is the protein accumulation level of SIZ1g higher than that of SIZ1c?

Thanks for the question. Higher protein accumulation was also our original hypothesis. However, we performed WB analysis of the two constructs using transient expression and found that the protein level of SIZ1g-GFP is comparable to that of SIZ1c-GFP. Although no clear difference in expression levels was detected by immunoblotting under these conditions, we still cannot rule out the possibility that differential expression contributes to the stronger cell death phenotype induced by SIZ1g, as dead cells may no longer contribute to the detectable protein pool. Uncharacterized splicing variants of SIZ1 may also contribute to this phenotype; but the causal factor behind is unclear and is beyond the focus of the current study.

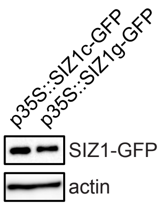

4. Line 274: Does the spontaneous necrosis observed in the siz1 mutant affect the accumulation of MAC proteins?

The analysis of MAC proteins shown in Fig. 5c (new Fig. 5d) was performed using 10-day-old seedlings. At this stage, the *siz1-2* mutant does not yet exhibit spontaneous necrosis.

5. In Figure 3f, after pathogen treatment, MAC3B-mediated condensates were observed in guard cells. Do similar condensates occur in non-guard cells? Does SIZ1 undergo phase separation in non-guard cells as well?

MAC3B condensates are not exclusive to guard cells and can be observed in other cell types, such as leaf epidermal cells (Jia et al., 2023, *Cell Host & Microbe*) and root cells after pathogen-induced immune induction (seedling co-culturing, see images below, left panel). However, we note a quantitative difference: the population of cells forming MAC condensates is significantly higher and easier to be recorded in guard cells compared to other tissue types following pathogen treatment. This observation suggests that guard cells may have a heightened responsiveness or a lower threshold for immune condensate formation.

In addition, co-condensation between SIZ1 and MAC3B also occurs in Arabidopsis leaf epidermal cells upon infection with Pst (image below, right panel).

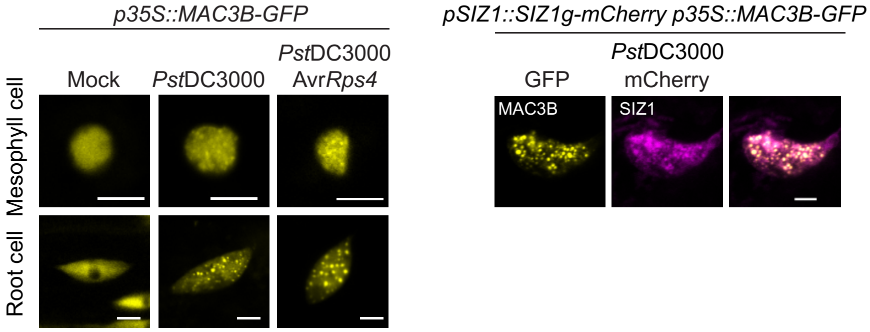

6. In Figure 3f, pathogen-induced phase separation of SIZ1 was observed in plants co-expressing MAC3B. Can pathogen infection alone trigger SIZ1 condensate formation in plants overexpressing SIZ1 only (without MAC3B co-expression)?

That is a good question. In fact, SIZ1 forms spontaneous nuclear condensates in transgenic lines with high SIZ1 expression, even in the absence of MAC co-expression (WT background; representative images shown on the right). Notably, these condensates are present in nearly all SIZ1–GFP lethal lines. These observations are consistent with the proposed model that elevated SIZ1 levels promote the hyper-SUMOylation of MAC proteins, enhancing their stability and subsequently driving MDNC formation and cell death. This data is now included in the new Extended Data Figure 3e.

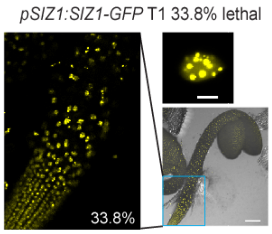

As noted in the text, we used a low SIZ1 expression line for the pathogen infection assays, which does not form spontaneous condensates under basal conditions. In this context, robust formation of large SIZ1 condensates upon pathogen infection is more difficult to detect in the absence of additional MAC3B co-expression, although such condensates can occasionally be observed. This is likely due to reduced efficiency of MAC SUMOylation at lower SIZ1 levels, possibly resulting in smaller, nanoscale condensates that fall below the detection limit of conventional microscopy even if they do form. Nevertheless, co-expression of MAC3B markedly enhances SIZ1–MDNC condensation, supporting the idea that SIZ1-dependent MAC SUMOylation constitutes a self-reinforcing process.

7. In Figure 3h, the condensate pattern (single fluorescent dot) appears different from that shown in Figure 3e (multiple fluorescent dots). Please clarify or discuss this discrepancy.

Nice observation. Indeed, in our tobacco transient expression assays, MAC5A formed condensates that exhibited heterogeneity in both size and number (see examples below). Notably, we frequently observed cells containing only one or two enlarged condensates. This observation supports the dynamic and fusion-prone nature of MAC condensates.

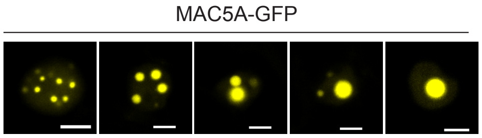

8. In Figure 4c, a p35S::KA120–YFP transgenic control is missing and should be included for comparison.

The new data with requested control is included in the Fig. 4c.

9. In Figure 5b and Extended Data Figure 5a, do the ubiquitination levels of MAC3B and MOS4 change in the siz1 mutant background? A key mechanistic question is how SIZ1-mediated SUMOylation interferes with MAC5A ubiquitination to enhance its stability. Additional genetic and biochemical evidence—such as constructing MAC5A point mutants defective in SUMOylation—would strengthen the conclusion that SUMO modification affects MAC5A ubiquitination and stability.

We predicted potential SUMOylation sites in two major MAC proteins investigated in this study, MAC5A and MAC3B, using SUMO-GPS and SUMOplot™. Four lysine residues with the highest prediction scores were mutated to arginine in each protein. For MAC5A, this quadruple mutation MAC5A^{4KR} almost completely abolished SUMOylation compared to the wild-type protein (Figure below, panel a). The MAC5A^{4KR} mutant exhibited an obvious change in condensation capacity: while

the wild-type protein localized exclusively to the nucleus and nuclear condensates, the mutant failed to form condensates (panel. b). This result supports our hypothesis that SUMOylation promotes the formation of MAC-associated nuclear condensates. The data were added to new Extended Data Figure 5e and 5f.

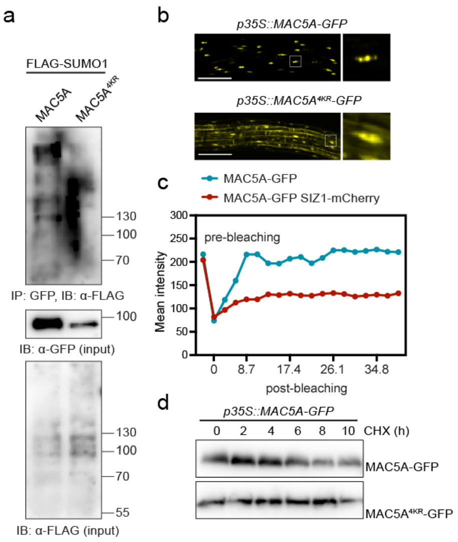

Our current model also indicates that MAC SUMOylation leads to enhanced multivalent interactions that promotes/stabilizes MAC condensation (Fig. 5e). Indeed, FRAP analysis showed that coexpression of SIZ1 markedly reduced the exchange rate of MDNC-localized MAC5A with the surrounding nucleoplasmic pool (panel c), suggesting that SUMOylated MAC is more stably incorporated or interlocked within the condensates. We hypothesize this is one of the mechanisms to reduce the spontaneous MAC ubiquitination and degradation. The data was added to new Extended Data Figure 5g.

Regarding the SUMOylation-dependent regulation of MAC ubiquitination, a full mechanistic dissection is challenging and likely confounded by multiple factors. First, SUMOylation may interfere with MAC ubiquitination. For example, contrary to our expectation, the MAC5A^{4KR} mutant exhibited increased stability compared with the wild-type protein (panel d). Because lysine residues can serve as targets for both SUMOylation and ubiquitination, and these modifications may exert opposing effects on MAC5A stability, their dual usage complicates interpretation of results obtained with MAC^KR mutants. This highlights a potential interplay between SUMOylation- and ubiquitination-dependent regulation of MAC activity that warrants future investigation.

We also assessed ubiquitination levels of MAC3B in *p35S::MAC3B-GFP, p35S::MAC3B-GFP/siz1-2,* and *p35S::MAC3B^{4KR}-GFP* plants. However, loss of SIZ1 did not significantly alter MAC3B ubiquitination (Figure on the right). Notably, MAC3B functions as an E3 ubiquitin ligase and is capable of auto-ubiquitination in vivo, further complicating precise attribution of ubiquitin signals. This represents a second factor that limits clear mechanistic resolution between these two modifications. Nevertheless, together with our observation that SIZ1-mediated effects on ubiquitination differ among other MAC proteins, these findings suggest that the interplay between SUMOylation and ubiquitination within the MAC complex is protein-specific and mechanistically complex.

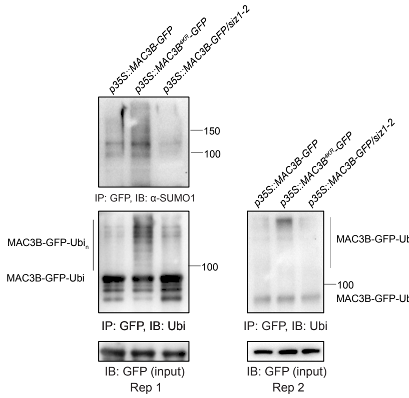

10. In Extended Data Figure 1d, were there any pSIZ1g::SIZ1–GFP/siz1 complementation lines that displayed necrosis? If so, is this phenotype correlated with the expression level of SIZ1–GFP?

We also observe necrosis in some of the *pSIZ1g::SIZ1–GFP/siz1* lines, specifically those with strong transgene expression. Furthermore, the severity of the necrotic phenotype was directly correlated with the expression level of the *SIZ1–GFP* transcript. This correlation reinforces our central finding that the autoimmune phenotype is a specific, dosage-dependent effect of SIZ1 accumulation and not a general artifact of the genetic background.

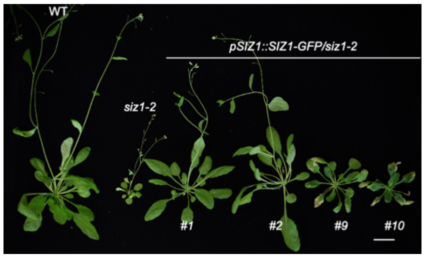

11. In Extended Data Figure 1j, SIZ1 mRNA levels increased under all tested treatments. Was a corresponding increase observed at the protein level?

Because a corresponding proteomics dataset is not available, we do not have information of the SIZ1 protein level under these specific biotic stress conditions at this moment.

12. In both SIZ1 overexpression and knockout backgrounds, how do MAC-dependent nuclear condensates (MDNCs) differ in number or persistence?
13. Do the nuclear condensates shown in this study exhibit fluorescence recovery after photobleaching (FRAP), indicating liquid-like behavior?

We found that pathogen induced MDNC formation and abundance are not significantly altered in the *siz1* mutant compared with WT. Nevertheless, MAC protein stability is clearly reduced in the absence of SIZ1, as shown in Fig. 5, suggesting that MDNC stability or persistence is likely compromised in the *siz1* mutant. We need to keep in mind that the interpretation of MDNC behavior in the *siz1* background is intrinsically complicated, as loss of SIZ1 itself activates immune responses through additional pathways. Such immune activation could enhance MDNC formation or persistence via mechanisms independent of the current study.

On the other hand, we quantified the MAC5A condensates number with or without overexpressing SIZ1-mcherry in tobacco transient expression system. When MAC5A is expressed alone, nearly 40% of cells exhibited only one or two enlarged condensates. In contrast, co-expression with SIZ1-mCherry led to a significant increase in condensate number per cell (see results on the left).

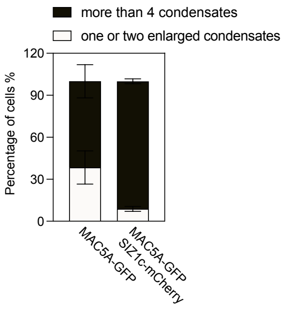

As mentioned before in Point 9, using FRAP, we found that the MAC5A fluorescence recovery was significantly reduced upon overexpression with SIZ1, indicating that SUMOylated MAC is more stably incorporated or interlocked within the condensates, consistent with the proposed model.

Together, these results demonstrate that overexpression of SIZ1 influences both the morphology (size and number) and the dynamic properties of MAC5A condensates.

14. The reviewer is also interested in whether the necrosis-inducing function of SIZ1 is conserved across species. Given the high evolutionary conservation of SIZ1, it would be worthwhile to examine whether overexpression of NbSIZ1 in Nicotiana benthamiana also triggers cell death, as observed in Arabidopsis.

This is an excellent question regarding the evolutionary conservation of SIZ1's function. Indeed, this specific experiment has been addressed in the field. As reported by Liu et al. (*New Phytologists*, 2022, 235: 1599), overexpression of *NbSIZ1* in *Nicotiana benthamiana* did not trigger cell death. This intriguing discrepancy suggests that while the SUMO E3 ligase SIZ1 is structurally conserved, its role in immune regulation and its capacity to induce cell death upon overexpression may have diverged between Arabidopsis and Nicotiana species, or may require specific, lineage-dependent interaction partners. This represents a fascinating area for future comparative studies.
